# Metallic nanomedicine in cancer immunotherapy

**DOI:** 10.1016/j.apsb.2025.07.017

**Published:** 2025-07-14

**Authors:** Shixuan Li, Xiaohu Wang, Huiyun Han, Shuting Xiang, Mingxi Li, Guangyu Long, Yanming Xia, Qiang Zhang, Suxin Li

**Affiliations:** a Department of Pharmaceutics, Jiangsu Key Laboratory of Drug Design and Optimization, State Key Laboratory of Natural Medicines, China Pharmaceutical University, Nanjing 211198, China; b State Key Laboratory of Natural and Biomimetic Drugs, School of Pharmaceutical Sciences, Peking University, Beijing 100191, China

**Keywords:** Metallic nanomedicine, Immune response, Cancer immunotherapy, Tumor microenvironment, Drug delivery system, Nanotechnology, Clinical application, Biosafety

## Abstract

Immunotherapy has become a pivotal modality in clinical cancer treatment. However, its effectiveness is limited to a small subset of patients due to the low antigenicity, impaired innate response, and various adaptive immune resistance mechanisms of the tumor microenvironment (TME). Accumulating evidence reveals the critical roles of metal elements in shaping immunity against tumor progression and metastasis. The marriage of metalloimmunotherapy and nanotechnology further presents new opportunities to optimize the physicochemical and pharmacokinetic properties of metal ions in a precise spatiotemporal control manner. Several metallodrugs have demonstrated encouraging immunotherapeutic potential in preliminary studies and are currently undergoing clinical trials at different stages, yet challenges persist in scaling up production and addressing long-term biosafety concerns. This review delineates how metal materials modulate biological activities across diverse cell types to orchestrate antitumor immunity. Moreover, it summarizes recent progress in smart drug delivery-release systems integrating metal elements, either as cargo or vehicles, to enhance antitumor immune responses. Finally, the review introduces current clinical applications of nanomedicines in metalloimmunotherapy and discusses potential challenges that impede its widespread translation into clinical practice.

## Graphical abstract

This review delineates how metal elements modulate biological activities across diverse cell types to orchestrate onco-immunity, and highlights the integration of metalloimmunology and nanotechnology in advancing antitumor therapy.

**Figure:**
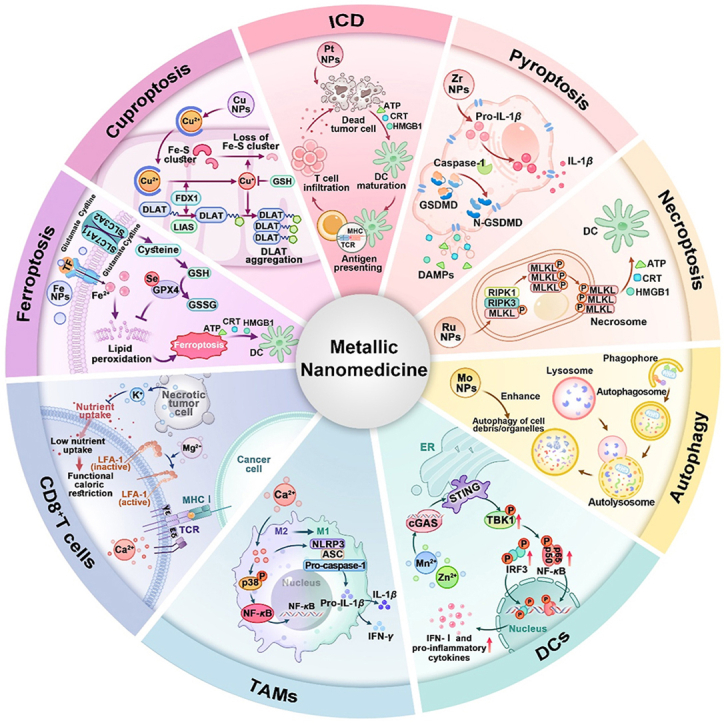

## 1 Introduction

Metal elements, accounting for approximately 2.5% of total body mass, play indispensable roles in sustaining human life and health. The medicinal properties of metal substances have been recognized since ancient times. For example, ancient Greeks utilized iron-rich rust to treat ailments, while cinnabar containing mercury (Hg) was used in ancient China to soothe nerves. In modern medicine, the discovery of platinum (Pt) as an antibacterial agent by Rosenberg and his colleagues in 1965 propelled the field of metallotherapeutics^1^^,^^2^. Since then, metal-based medicines have been extensively investigated and applied in the treatment of multiple diseases, particularly cancer, where cisplatin stands out as the first and most renowned metallodrug approved for clinical use^3^.

Immunotherapy has marked a significant leap forward in cancer treatment over the past decade^4^. For an effective antitumor immune response to occur, a sequence of coordinated events needs to proceed, including the release of tumor antigens, their presentation by antigen-presenting cells (APCs), priming of effector cells and their infiltration into tumors, and subsequent recognition and elimination of tumor cells^5^^,^^6^. This process in turn generates fresh tumor antigens to initiate a new cycle of antitumor immunity. Therapeutic approaches such as neoantigen vaccines, functional cytokines, adoptive cell transfer, and immune checkpoint inhibitors have been developed to target specific phases of the cancer-immunity cycle^7^, ^8^, ^9^, ^10^, ^11^. Compared to traditional chemotherapies, immunotherapies harness the body's own immune system to combat cancer, potentially improving safety and fostering long-term protective memory^12^. However, tumors can adapt by evolving various resistance mechanisms to evade or suppress antitumor immunity, particularly in advanced tumor stages, where these mechanisms become increasingly intricate and diverse^13^, ^14^, ^15^. Therefore, there is an urgent need for innovative strategies to bolster and sustain antitumor immune responses, alongside the development of effective drug combinations that target multiple stages of the cancer-immunity cycle.

Metallic elements are endogenous elements in the human body and have demonstrated their essential roles in shaping the immune system^16^. For example, potassium (K^+^), calcium (Ca^2+^), and magnesium (Mg^2+^) are responsible for the stemness and cytotoxic functions of T and NK cells^17^, ^18^, ^19^. Sodium (Na^+^), zinc (Zn^2+^), and manganese (Mn^2+^) activate pattern recognition receptors (PRRs) like Toll-like receptors (TLRs), stimulator of interferon genes (STING), and nuclear factor kappa-B (NF-*κ*B) as danger signals for immune recognition^20^, ^21^, ^22^, ^23^. Iron (Fe^2+/3+^) and copper (Cu^2+^) can induce tumor cell death through immunogenic mechanisms, triggering the release of “eat me” signals and recruiting functional immune cells, thereby transforming the TME from immune-suppressive (“cold”) to immune-responsive (“hot”) phenotype^24^, ^25^, ^26^, ^27^. Therefore, these endogenous metallic elements are often regarded as safer and more biologically compatible (when administered at optimal doses and delivered precisely) compared to artificial immune stimulators. Additionally, many metallic elements function as essential nutrients that help maintain biological homeostasis and strengthen the immune system, preparing it to combat cancer effectively. For example, Fe^2+^ is involved in transporting oxygen and supporting tissue respiration, while Zn^2+^ plays an important part in gene regulation in these organisms^28^. Na^+^ and K^+^ are key in maintaining acid-base homeostasis in body fluids and facilitating electron transfer in immune cellular metabolism^29^, ^30^, ^31^. Furthermore, about 30% of enzymatic reactions rely on metal cofactors such as Zn^2+^, Mg^2+^, and Cu^2+^ ^32^. Despite their promise, concerns regarding *in vivo* adsorption, distribution, metabolism, and excretion (ADME) processes, as well as long-term toxicity risks of metal substances, limit their broad clinical application^33^. Engineering metal materials into suitable drug formulations could unlock their inherent therapeutic potential.

Beyond direct immunomodulatory actions, metal substances often exhibit substantial surface area and abundant functional groups (or vacant electron orbitals) and can be further tailored to exhibit specific structural or functional features, such as nano-size or photo/thermal/magnetic responsiveness^34^^,^^35^. These contributions make them ideal candidates as vehicles for on-demand delivery and release of other immunomodulators while also serving as adjuvants in combination therapies. These effects foster robust immune responses and provide new insights into the design of the state-of-art metallic nanomedicines for cancer immunotherapy, particularly in the context of drug combination strategies aimed at achieving synergistic effects.

This review highlights the immunological significance of metallic nanomedicines across diverse cell types within the TME, including their direct effects on immune cells, their role in modulating the extracellular matrix or alleviating immunosuppressive features within tumor tissues, and their indirect effects in inducing immunological tumor cell death. It also introduces recent laboratory and preclinical studies on the utilization of metal-based nanoparticles in cancer immunotherapy. Furthermore, it summarizes metallic nanomedicines currently under different stages of clinical trials and discusses translational hurdles, aiming to guide the future development of metalloimmunotherapy strategies.

## 2 Metallic nanomedicines induce tumor cell death

**Figure 1:**
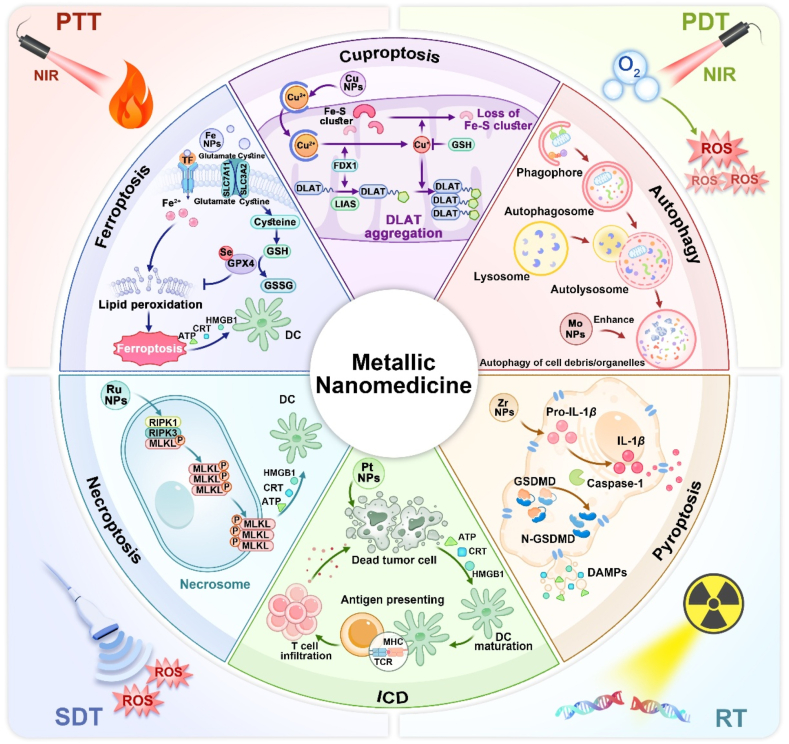
Metallic nanomedicines induce various modes of immunogenic tumor death.

Metal ions play critical roles in various fundamental biological processes including membrane excitability, metabolic regulation, enzymatic catalysis, signal transduction, redox balance, and energy supply^36^. The intracellular concentration of metal ions is tightly regulated, and even slight shifts can disrupt cellular hemostasis and eventually leading to tumor cell death^37^^,^^38^. Typically, the death of tumor cells can be classified into accidental cell death (ACD) and regulated cell death (RCD)^39^. Unlike ACD, which occurs due to acute damage or stress beyond a cell's regulatory capabilities^40^. RCD is a controlled form of cell death governed by genetic mechanisms and structured signaling pathways, often linked to immune responses^41^^,^^42^. In 2018, based on the biochemical mechanisms and morphological features, the Nomenclature Committee on Cell Death (NCCD) further categorized RCD into ferroptosis, cuproptosis, pyroptosis, autophagy, necroptosis, immunogenic cell death, disulfidptosis, alkalosis, oxeiptosis, parthanatos, NETotic cell death, and others^43^^,^^44^. Next, we delve into the specific roles of metallic nanomedicines in inducing several representative types of RCD and their implications for immunotherapy (Fig. 1 and Table 1) 45, 46, 47, 48, 49, 50, 51, 52, 53, 54, 55, 56, 57, 58, 59, 60, 61, 62, 63, 64, 65, 66, 67, 68, 69, 70, 71, 72, 73, 74, 75.

**Table 1:** Metallic nanomedicines induce various modes of immunogenic tumor death.

Category	Metal element	Nanomedicine	Mechanism	Tumor model	Ref.
Ferroptosis	Fe	ssPPE_Lap_@Fe-TA	Fe^3+^-mediated Fenton reaction for systemic antitumor immunity	CT26	45
	Fe	PAD@MS	Inducing LPO accumulation for DC maturation and T cell activation	MC38/CT26	46
	Cu	Cu_2−x_Se/ZIF-8@Era-PEG-FA	Catalyzing Cu ^+^ to produce O_2_ for ferroptosis, leading to T cell activation	4T1	47
	Ir	IrFc1	Inducing LPO to promote ferroptosis for T cell activation	TNBC	48
Cuproptosis	Cu	NP@ESCu	Promoting mitochondrial Cu^+^ accumulation for DC maturation	MB49	49
	Cu	PTC	Inducing cuproptosis for immune memory effects	4T1	50
	Cu	^Cu/AP^H-M	Decomposing H_2_O_2_ in the TME into O_2_ for cuproptosis	CT26	51
	Cu	BSO-CAT@MOF-199 @DDM	Reducing Cu^2+^ to toxic Cu ^+^ by FDX1, facilitating T cell infiltration	GL261	52
Pyroptosis	Zr	ZrNPs	Inducing ROS for caspase-1 activation and GSDMD cleavage, activating DC	4T1	53
	Fe/Mn	FeMn@R@H	Initiating Fenton-like reactions for ROS-mediated pyroptosis	4T1	54
	Ca	CaNM	Inducing mitochondrial Ca^2+^ overload for caspase-3 activation, promoting DC maturation and T cell activation	4T1	55
	Au/Ag/Bi	Au@AgBiS_2_	Inducing caspase-3 activation and releasing DAMPs	4T1	56
	Fe/Zn	FZOH	Activating caspase-1/GSDMD-dependent pathway	4T1	57
Autophagy	Mo	MoO_3-x_	Promoting cytotoxic autophagy and release of tumor antigens	HepG2	58
	Ti	NBP/TiO_2_	Blocking autophagosome fusion and inhibiting lysosomal degradation	U-87 MG	59
Necroptosis	Fe/Pd	FPS-LNPs	Combining necroptosis and ferroptosis, triggering DAMP and antigen release	4T1	60
ICD	Gd/Zn	Gd-MOF-5	Downregulating phosphatidylserine signal and promoting ER stress	4T1/MCF-10A	61
	Ce/Cu	CBPNs@HA	Amplifying oxidative stress and inducing tumor-specific antigen release	4T1	62
ICD (PTT)	Cu	IL@H-PP	Sensitizing PTT effects	4T1	63
	Au	AuND	Generating PTT effects for DC maturation and cytokine secretion	K7M2	64
	Au/Pt	PMIA	Plasmon-mediated catalytic reactions for ROS generation and DNA damage	4T1	65
ICD (PDT)	Au/Mn	AuNC@MnO_2_	Sensitizing PDT effects	4T1	66
	Ca/Cu/Mn	CCMH	Sensitizing PDT effects and Ca^2+^ overload	4T1/CT26	67
ICD (SDT)	Ca	TiO_2_@CaP	Sensitizing SDT effects for mitochondrial dysfunction	4T1	68
	Ir	HSA@Ir-CA	Disrupting ER and amplifying ICD-initiated immune responses	4T1	69
ICD (RT)	Fe	OXA/Fe NPs	Amplifying RT effects by inducing DNA damage	CT26	70
	Gd	H@Gd-NCPs	Combining ROS generation and GSH depletion for RT-induced ICD effects	CT26/4T1	71
	Hf	UiO@MnS	Enhancing radiotherapy to increase antitumor immunogenicity	4T1/MCF-7	72

Due to space constraints, we regret that we cannot include all relevant cases here; however, they are available in other related reviews^73^, ^74^, ^75^. DC, dendritic cells; DAMP, damage-associated molecular pattern; ER, endoplasmic reticulum; FDX1, ferredoxin 1; GSH, glutathione; ICD, immunogenic cell death; LPO, lipid peroxidation; PDT, photodynamic therapy; PTT, photothermal therapy; SDT, sonodynamic therapy; RT, radiation therapy; ROS, reactive oxygen species.

### 2.1 Ferroptosis

Ferroptosis is an iron-dependent form of RCD characterized by overload of iron ions and accumulation of lipid peroxidation (LPO)^76^. During ferroptosis, unstable reactive Fe^2+^ ions accumulate within the cell and interact with polyunsaturated fatty acids (PUFAs), leading to the formation of lipid peroxides^77^. These products generate intracellular oxidative stress, disrupt plasma membrane permeability, and trigger the release of antigens and other immunocontents, eventually enhancing both antigenicity and adjuvanticity in the TME for an effective antitumor immune response^78^.

Multiple nanotherapeutic approaches have been developed to deliver iron ions into tumor cells to induce ferroptosis. For example, Dai et al.^45^ engineered a rigid metal-polyphenol shell-modified nanodevice that underwent discomposing in the acidic endolysosomes and released iron ions within tumor cells, triggering the Fe^3+^-mediated Fenton reaction [Fe^2+^ + H_2_O_2_ → Fe^3+^ + (OH)^–^ +·OH]. The resulting hydroxyl radicals damaged tumor cells *via* the PUFA-LPO mechanism, which led to the generation of several damage-associated molecular patterns (DAMPs), including calreticulin (CRT) exposure, high mobility group protein 1 (HMGB1) release, and adenosine triphosphate (ATP) secretion^79^^,^^80^. These DAMPs acted as “eat me” signals, promoting infiltration and maturation of APCs by binding to PRRs on the cell surface, augmenting the expansion and differentiation of tumor-specific cytotoxic CD8^+^ T cells^81^. Furthermore, the disulfide-containing polyphosphoester in the core of the nanosystem efficiently depleted GSH *via* a disulfide-thiol exchange reaction, leading to the inactivation of glutathione peroxidase 4 (GPX4), the only antioxidant enzyme that protects the cell membrane from LPO by converting lipid hydroperoxides into non-toxic lipid alcohols^82^^,^^83^. This depletion of GSH and inactivation of GPX4 further potentiated ferroptosis, enhancing antitumor immune responses at a relatively low Fe dose.

**Figure 2:**
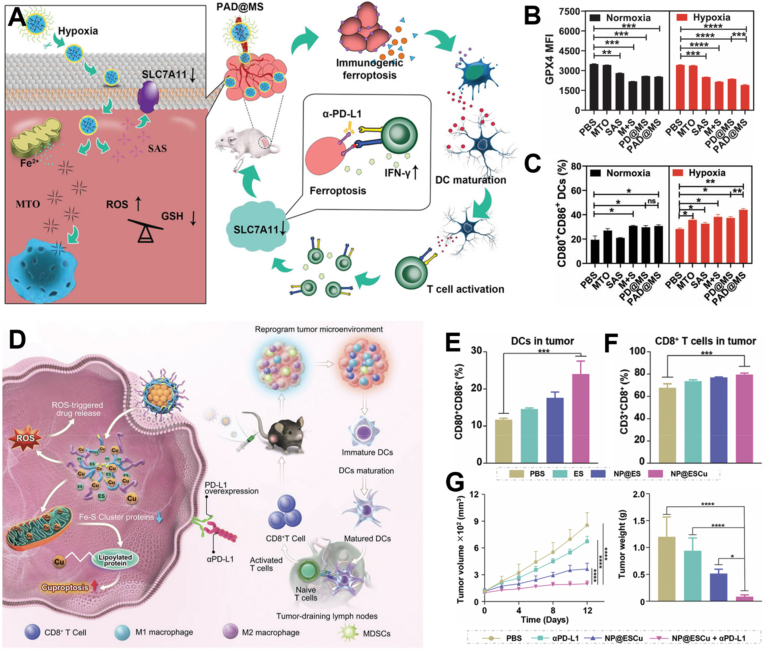
Representative examples of metallic nanomedicines inducing tumor death. (A) A hypoxia-response nanoplatform (PAD@MS) induced immunogenic ferroptosis by suppressing GPX4 and promoting ROS accumulation. (B) Flow cytometry analysis of GPX4 content within tumor cells after PAD@MS treatment. (C) Flow cytometry analysis of DC maturation after co-incubated with PAD@MS-pre-treated MC38 tumor cells. Reprinted with the permission from Ref. 46. Copyright © 2023, Wiley-VCH GmbH. (D) NP@ESCu induced cuproptosis by promoting the accumulation of Cu^2+^ in mitochondria *via* co-encapsulating copper and elesclomol. (E) The percentage of matured DC (CD80^+^CD86^+^) populations and (F) CD8^+^ T cell populations in tumors with various treatments. (G) Tumor growth curves and *ex vivo* tumor weight of mice with various treatments. Reprinted with the permission from Ref. 49. Copyright © 2023, Wiley-VCH GmbH.

The mechanisms through which metallic nanomedicines induce ferroptosis primarily involve the promotion of intracellular Fenton or Fenton-like reactions, inhibition of GPX4, and modulation of LPO levels^82^^,^^84^. The relationship between ferroptosis and immunotherapy is often bidirectional^85^. Ferroptosis activates antitumor immunity by triggering the release of immunogenic substances, and subsequently activating dendritic cells (DCs) and CD8^+^ T cells against cancer. Meanwhile, these immune responses can further promote LPO and potentiate ferroptosis of tumor cells. For example, Cai et al.^46^ devised a coordination nanoparticle that released Fe^3+^ and sulfasalazine (SAS) within the hypoxic TME (Fig. 2A–C). In the system, Fe^3+^ fostered reactive oxygen species (ROS) production while SAS caused 70% reduction in GPX4 expression by blocking the glutamate-cystine antiporter protein SLC7A11, synergistically enhancing LPO accumulation for enhanced immunogenic ferroptosis. The released “find-me” and “eat-me” immunostimulatory signals recruited DCs and elicited cytotoxic T lymphocyte responses for tumor infiltration. Interestingly, the interferon *γ* (IFN-*γ*) released by activated CD8^+^ T cells in turn enhanced ferroptosis by downregulating SLC7A11 expression, creating a positive feedback loop. While these ferroptosis inducers are potent and act rapidly, they may also pose significant toxic side effects; for instance, metal-based GPX4 inhibitors can impair the development and function of the nervous system and kidneys^86^. It is crucial to ensure tumor-specific activation of the Fenton response to minimize off-target toxicity to normal tissues^87^^.^

### 2.2 Cuproptosis

The cytotoxic effects of excess copper have long been recognized, yet the underlying mechanism remained unclear until Tsvetkov et al.^88^ proposed the concept of cuproptosis in 2022. Excess Cu^2+^ in mitochondria is reduced to the more toxic Cu ^+^ by ferredoxin 1 (FDX1)^89^, facilitating the lipidation of dihydrolipoamide S-acetyltransferase (DLAT) as well as inhibiting the formation of iron-sulfur (Fe–S) clusters, leading to a proteotoxic contingency^73^. However, existing copper ionophores have limited efficacy due to their short circulation half-life and poor tumor accumulation as small molecules^90^.

Guo et al.^49^ developed polymeric nanoparticles (NP@ESCu) co-encapsulating copper and elesclomol to enhance copper delivery into tumor cells (Fig. 2D–G). It elevated intracellular ROS for the liberation of copper and elesclomol, which then targeted mitochondria to induce dihydrolipoyl transacetylase aggregation and Fe–S cluster protein loss. As a result, NP@ESCu promoted robust cuproptosis and reshaped the immunosuppressive TME, as evidenced by the activation of pathways regarding the cytokine–cytokine receptor interaction, DC antigen processing and presentation, as well as T cell receptor signaling. Compared to elesclomol nanoparticles (NP@ES) alone, NP@ESCu significantly boosted the JAK–STAT and PI3K–Akt signaling, which are essential in regulating adaptive immune responses. Elevated copper levels also increased PD-L1 expression on tumor cells, rendering them more susceptible to immune checkpoint blockade therapy, effectively improving the response rate of *α*PD-L1.

Tumor cells can possess self-protective mechanisms against cuproptosis by spontaneously exocytosing copper ions^91^. Therefore, strategies that deliver copper into tumor cells while inhibiting the exocytosis negative feedback loop are crucial for enhancing therapeutic efficacy. Ning et al.^50^ engineered a platelet membrane-camouflaged cuprous oxide nanoparticle (Cu_2_O)/TBP-2 cuproptosis sensitization system (PTC) for this purpose. Upon internalization by tumor cells, PTC rapidly degraded to release copper ions, inducing lipoylated protein aggregation and Fe–S cluster protein loss. Meanwhile, the released TBP-2 quickly entered the cell membrane and inhibited copper efflux. PTC amplified cuproptosis by approximately 88% compared to single Cu_2_O nanoparticles, and further enhanced the proportion of CD62L^+^CD44^+^ subsets among CD8^+^ T cells, indicating a potent immune memory protection. As a form of cell death reliant on mitochondrial respiration, cuproptosis is highly influenced by oxygen levels^26^. Pei et al.^51^ devised a copper-loaded oxygen generator (^Cu/AP^H-M) to alleviate the hypoxic TME and deliver Cu^2+^ for enhanced cuproptosis. It elicited more robust immunogenic effects than single Cu nanoparticles, as evidenced by the upregulation of caspase-1, CRT, heat shock proteins (HSP70 and 90), and tumor necrosis factor *α* (TNF-*α*). ^Cu/AP^H-M also reversed the expression of PD-L1 in tumor cells, thus reducing the immunological escape of tumor cells. This cascade of immune events contributed to the activation of the antitumor immune cycle, inducing potent therapeutic effects against primary, abscopal, and recurrent tumors.

Above studies highlight that targeting negative feedbacks and compensatory mechanisms involved in cuproptosis could enhance the therapeutic efficacy of copper-based nanomedicines^49^, ^50^, ^51^. The field of cuproptosis remains in its early stages, with a limited understanding of the underlying molecular and biological mechanisms^92^. Key factors, such as the specific role of FDX1, the interaction of cuproptosis with other signaling pathways, and tumor resistance mechanisms during cuproptosis, require further exploration^26^. From a delivery perspective, modifying circulation or cellular pharmacokinetic properties may represent a promising strategy to sustain lethal intracellular copper concentrations, thereby promoting effective antitumor immune responses^93^. However, it is important to recognize that copper metabolism plays a dual role in both tumorigenesis and cancer therapy^94^. Copper contributes to tumor cell energy production through its involvement in mitochondrially encoded cytochrome C oxidase (MT-CO) and activates various oncogenic signaling pathways, including RAS–RAF–MEK–ERK and PI3K–AKT–mTOR^95^. Striking a balance between the therapeutic benefits of cuproptosis and the risks of inadvertently promoting cuproplasia in tumor cells presents a substantial challenge.

### 2.3 Pyroptosis

In 2001, Cookson et al.^96^ proposed the concept of pyroptosis as a novel cell death mechanism. Pyroptotic cells are characterized by swelling and membrane blebbing before eventual rupture, distinguishing them from apoptotic cells^97^. Metal ions overload, such as Ca^2+^, Na^+^, K^+^, Zn^2+^, and Bi^3+^, has been reported to induce pyroptosis^98^^,^^99^. During the process, inflammatory caspases cleave gasdermin (GSDM) family proteins to generate the N-terminal domain (GSDM-N), which interacts directly with membrane lipids to form pores in the plasma membrane, leading to the leakage of intracellular immunocontents^100^. Consequently, pyroptosis elicits immune responses more readily than the silent death of apoptosis. Zheng et al.^101^ designed a Ca^2+^ nanomodulator that degraded within acidic tumor cells and triggered mitochondrial Ca^2+^ accumulation. The Ca^2+^ overload subsequently facilitated cytochrome C release to activate caspase-3 for GSDM-E cleavage. This process effectively induced pyroptosis and released a large number of inflammatory molecules, leading to increased DC maturation and CD8^+^ T cell activation by 2.5-fold and 3.8-fold, respectively.

Metal ions such as Fe^2+^, Mn^2+^, and zirconium (Zr^2+^) have also been reported to induce pyroptosis through different mechanisms^53^^,^^99^. For instance, Feng et al.^54^ developed an acid-responsive Fe/Mn bimetal-organic framework nanosystem designed to release Fe^2+^ and Mn^2+^ within tumor cells and produce ROS *via* Fenton and Fenton-like reactions. These excess ROS not only caused direct oxidative damage to cell membranes and organelles, but also significantly activated the NOD-like receptor thermal protein domain associated protein 3 (NLRP3) inflammasome, which processed the activation of caspase-1 and converted pro-interleukin-1*β* (pro-IL-1*β*) and pro-IL-18 into their bioactive forms. These cytokines further cleaved GSDM-D proteins into functional domains, resulting in robust pyroptosis and subsequent release of pro-inflammatory cytokines and immunogenic components from tumor cells. As a result, the Fe/Mn bimetal nanosystem promoted DC maturation and antigen presentation compared to single metal-based counterparts.

The GSDM family comprises several members, such as the D-form and E-form as mentioned above ^102^. It would be intriguing to explore whether different metal ions preferentially interplay with specific forms and influence distinct pyroptosis efficacy. Furthermore, while inducing pyroptosis generally enhances the antitumor immune response, excessive pyroptosis can contribute to the onset of autoimmune diseases and nephrotoxicity^103^. Integrating electromagnetic or photo-guided metal-based nanomaterials could be an effective strategy to improve the spatiotemporal control of pyroptosis inducers, enabling precise on-demand cancer therapy^104^.

### 2.4 Autophagy and necroptosis

Autophagy is a natural cellular process that degrades unnecessary or damaged organelles within the cell^105^. Metallic materials, including gold nanoparticles, iron oxide (Fe_3_O_4_), and cerium dioxide (CeO_2_), were reported to exert influence on autophagy through diverse mechanisms such as elevating oxidative stress, disrupting certain signaling pathways, inducing mitochondrial damage, and triggering endoplasmic reticulum (ER) stress^106^, ^107^, ^108^. The role of autophagy in cancer progression exhibits complex and sometimes contradictory regulatory effects. On the one hand, autophagy supports tumor cell survival by preserving genomic stability, eliminating redundant proteins and damaged organelles, and supplying nutrients and energy, especially in the early stages of tumor igenesis^109^. Based on this, Wang et al.^108^ synthesized porous CeO_2_ nanorods that inhibited the activation of PI3K/AKT and p38MAPK pathways in tumor cells, thereby blocking protective autophagy and promoting strong antitumor responses. On the other hand, excessive activation of autophagy can initiate the autophagic cell death pathway and enhance the release of immunogenic factors in dying tumor cells. He et al.^58^ developed MoO_3-x_ nanowires capable of generating ROS and triggering cytotoxic autophagy. *In vitro* and *in vivo* experiments demonstrated that these nanowires facilitated the release of tumor antigens, thereby promoting APC activation and infiltration of effector T cells. Moreover, the combination of MoO_3-*x*_ nanowires and PD-1 blockade could inhibit the infiltration of regulatory T cells and promote the polarization of macrophages into tumor-killing M1 phenotypes, resulting in mitigated immunosuppression in the TME and complete tumor eradication. In summary, autophagy appears to act as a double-edged sword in tumor progression, exerting both promotional and suppressive effects on cancer immunotherapy^110^. The effects likely depend on factors such as tumor type and stage, as well as the dosage and physiochemical properties of therapeutic agents^111^. Further investigation is required to elucidate which role predominates in different contexts and how to manipulate autophagy for optimal therapeutic outcomes.

Unlike autophagy, necroptosis is typically triggered by death receptors, including but not limited to the tumor necrosis factor receptors (TNFR), TLRs, and nucleic acid sensors^112^. Stimulation of these receptors drives the assembly of a necrosome comprising receptor-interacting protein kinase (RIPK1/3) and mixed lineage kinase domain-like (MLKL)^113^. Metal-based chemotherapeutic agents have demonstrated the capability to induce tumor necroptosis^114^. Necroptotic cells exhibit compromised plasma membrane integrity and are phagocytosed more slowly than apoptotic cells, providing extended time for releasing antigens and DAMPs that stimulate subsequent immunogenic activities^115^. However, the use of metal-based nanoparticles to induce necroptosis is still in its infancy. Mechanisms underlying the diverse necroptotic pathways activated by different metal substances and their efficiencies remain to be explored. Additionally, when nanoparticles trigger necroptosis, it is crucial to consider the potential activation of protective autophagy, which could prevent tumor cells from death^116^. Therefore, combining metal inducers of necroptosis with autophagy inhibitors may enhance antitumor therapeutic efficacy^117^.

### 2.5 ICD

Immunogenic cell death (ICD) is a subtype of RCD that elicits an effective adaptive immune response by generating multiple tumor-specific and tumor-associated antigens from cell debris^118^. These antigenic epitopes are subsequently taken up, processed, and presented by APCs to effector T cells. As the first metal-based antitumor drug in modern medicine, oxaliplatin directly binds to intracellular DNA within the nucleus, leading to cross-linking of DNA strands, which ultimately results in DNA breaks and leakage^3^. In addition to its intrinsic anti-proliferative properties, the resulting DNA damage acts as a DAMP increasing tumor immunogenicity. Nanoparticles can facilitate the targeting capability of Pt compounds toward tumor cell nucleus, thereby augmenting antitumor immunity^119^^,^^120^. Other metallic nanomedicines containing Ru and iridium (Ir) have also shown promise in inducing ICD by disrupting intracellular iron metabolism, interfering with energy metabolic pathways, and generating oxidative stress within tumor cells, which collectively enhanced both antigenicity and adjuvanticity within the TME^121^, ^122^, ^123^.

Certain metal nanoparticles, particularly those made from precious metals like gold and silver, exhibit a unique localized surface plasmon resonance (SPR) effect^124^^,^^125^. This effect results in exceptional photothermal conversion efficiency^126^. When the frequency of incoming light aligns with the SPR frequency of these nanoparticles, it leads to intense light absorption and scattering, causing collective oscillations of free electrons on their surfaces^127^. The absorbed light energy is then converted into heat through a non-radiative relaxation process, significantly elevating the local temperature to facilitate tumor ablation^128^. This technique, known as photothermal therapy (PTT), offers a minimally invasive approach to eliminate tumor cells while also enhancing tumor immunogenicity^63^^,^^129^. Yan et al.^130^ engineered a semiconductor nanomaterial consisting of copper sulfide, which achieved localized tumor ablation through PTT, releasing antigens and DAMPs from the tumor cell residues. Compared with the single laser treatment group, it significantly promoted intratumoral infiltration of CD8^+^ T cells, leading to a 14-fold increase in tumor inhibition. The copper sulfide nanoparticles further synergized with protein tyrosine phosphatase non-receptor type 2 inhibition to completely abolish tumor growth.

Some photosensitizers can transition photons from their ground state to an excited state under near infrared (NIR) irradiation, transferring the excitation energy to oxygen molecules and promptly generating cytotoxic ROS^131^. This process, known as photodynamic therapy (PDT), also contributes to ICD effects^132^. However, its clinical utility is hampered by photobleaching and aggregation-caused-quenching (ACQ), which are common among most organic photosensitizers, resulting in a restricted diffusion radius of less than 0.02 μm and a short ROS lifespan under 0.04 μs^133^^,^^134^. To address this challenge, Li et al.^135^ developed Pt metallacycles capable of generating ROS within a 7 mm radius, with strong anti-ROS quenching ability. The Pt metallacycles facilitated deep-seated tumor immune responses with minimal adverse effects.

Metal ions with high dielectric constants can produce hydroxyl radicals through the piezoelectric effect induced by ultrasound, leading to the induction of sonodynamic therapy (SDT)^136^. Liang et al.^137^ utilized polypyridinal metal complexes (Ru(bpy)_3_) to produce singlet oxygen and sono-oxidize endogenous 1,4-dihydronicotinamide adenine dinucleotide under ultrasound stimulation in tumor cells, achieving therapeutic effects in deep tissues exceeding 10 cm and effectively eliminating tumors. However, the natural tendency of electron-hole recombination may reduce the efficacy of SDT. To address this challenge, Xu et al.^69^ introduced cholic acid-functionalized iridium nanosonosensitizers, which selectively accumulated in the ER and disrupted it precisely with *in situ* production of both type I and type II ROS upon ultrasound stimulation. Significant up-regulation of the expression of ER stress-related proteins, such as GRP78 and CHOP, and higher extracellular release of ATP and HMGB1, were observed upon sonication plus nanosonosensitizer treatment compared to single treatment.

Radiotherapy (RT) employs high-energy ionizing radiation to induce DNA damage in exposed cells and is widely used in clinical cancer treatment^70^^,^^138^. Metal ions with high atomic numbers such as gadolinium (Gd), bismuth (Bi), cerium (Ce), tungsten (W), and Hafnium (Hf) can enhance radiation energy deposition by emitting secondary electrons^139^. Huang et al.^71^ fabricated nanoscale coordination particles self-assembled from Gd and 5′-guanosine monophosphate as RT sensitizers (Gd-NCPs). Gd-NCPs effectively enhanced X-ray absorption and increased hydroxyl radical production. Incorporating Hemin into the system (H@Gd-NCPs) further endowed it with peroxidase-like properties to utilize overexpressed H_2_O_2_ in the TME, depleting GSH and amplifying RT-mediated oxidative stress. Notably, the combination of RT with H@Gd-NCPs demonstrated a significant abscopal effect against distant metastases, reaching a survival rate of 30% at the end of the study. In contrast, all the mice died in the RT monotherapy treatment group. This phenomenon was mediated through ROS production, which disrupted cell nucleus integrity for ICD, exposing tumor antigens and DAMPs. Flow cytometric analysis revealed that H@Gd-NCPs enhanced T cell infiltration into both primary and distant tumors, confirming their ability to sensitize RT and strengthen immune-mediated cancer effects.

As a genetically controlled cell death process, RCD not only plays a critical role in maintaining tissue homeostasis, but also potentially activates antitumor immune system by triggering various immune signaling cascades and releasing antigens, DAMPs, and pro-inflammatory factors^79^. The distinctions among RCD modalities (ferroptosis, cuproptosis, pyroptosis, autophagy, necroptosis, ICD, and others) are characterized by their triggering mechanisms, morphological features, and the specific signaling pathways involved^115^. However, recent studies suggested that their distinctions are not always so clear-cut. For instance, while excess Fe is known to induce ferroptosis while Cu accumulation causes cuproptosis, non-ferrous metals such as Mn, Co, Mo, and even Cu were also reported to promote ferroptosis by altering their valence states during oxidation^47^^,^^140^^,^^141^. Additionally, localized high temperatures generated by PTT is found to drive the assembly of inflammatory vesicles^142^. ROS produced by PDT can directly damage the lipid membrane of tumor cells, leading to lipid peroxidation and the onset of ferroptosis^143^. Zeng et al.^144^ reported Ir-based photosensitizers could activate both GSDM-associated pyroptosis and GPX4-related ferroptosis through KEAP1/NRF2/HO-1 axis. Although it sounds plausible to consider using a single metallic nanoparticle to activate multiple RCD mechanisms for combination therapy, previous research has indicated that activating autophagy pathways may generate resistance against necroptosis^113^. Therefore, understanding the intrinsic connections between these processes may be crucial for achieving true synergistic effects. A recent study demonstrated a novel approach by combining iron with carbonyl cyanide *m*-chlorophenyl hydrazone to engineer a nanocatalytic medicine, which regulated the transition from ferroptosis to pyroptosis within tumor cells, ultimately enhancing antitumor immune responses^145^. This study suggests that pyroptosis may be more immunogenic than ferroptosis, though the underlying mechanisms remain to be elucidated. Besides, is the influence of a single metal substance on multiple pathways also dose-dependent? Do different tumor types exhibit varying sensitivities to specific RCD modes? Identifying key molecular mechanisms that dictate pathway preference could ultimately influence cell fate^146^. In this case, pre-assessing genomic and proteomic profiles may help determine a patient's suitability for personalized medicine^147^.

## 3 Metallic nanomedicines modulate immune cells

**Figure 3:**
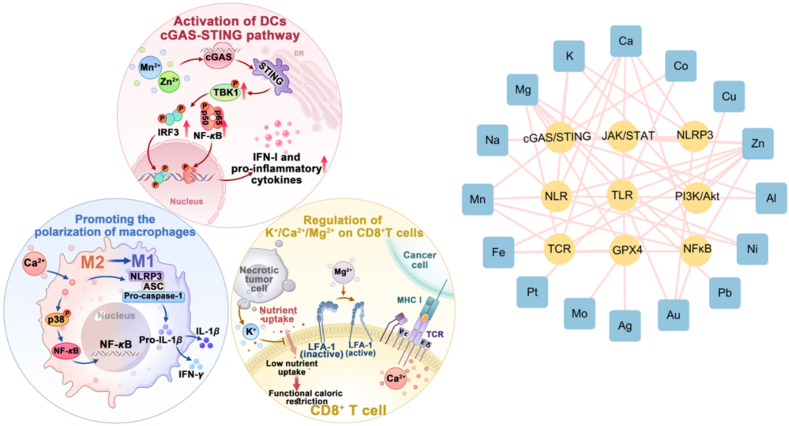
Metal components modulate the antitumor function of DC, macrophage, and CD8^+^ T cells through various immune signaling pathways.

In addition to inducing various immunologic modes of RCD in tumor cells for secondary immune responses, metal ions can also directly participate in the regulation of immune cell development and function (Fig. 3)^37^^,^^148^^,^^149^. Innate immune responses, such as phagocytosis for macrophages and natural cytotoxicity for natural killer (NK) cells, constitute the frontline defense against invading pathogens and tumor growth^150^^,^^151^. Moreover, the innate sensing of tumor antigens by APCs initiates antigen-specific and more powerful adaptive immune responses^152^. Certain metal ions have been identified to promote these immune sensing processes through stimulating various PRRs, such as TLRs (Ni, Co, Pb, Pt, Fe), STING (Zn, Mn, Co, Ca, Mg), NF-*κ*B (Fe, Mg, Zn), and NLRP3 (K, Ca, Na, Mn, Gd, Al)^149^^,^^153^^,^^154^. A key aspect of adaptive immunity involves the activation of the T cell receptor (TCR)–CD3 complex and subsequent cytotoxic signaling cascade in T cells, which is finely tuned by the influx of Ca^2+^, Mg^2+^, and Zn^2+^ ^155^^,^^156^. Additionally, the maintenance of T cell stemness and the duration of immune memory are closely associated with K^+^ levels^17^. Collectively, metal ions are essential in coordinating both innate and adaptive immunity against cancer. However, their intracellular access is often hindered by challenges such as aggregation, fast degradation, or the limited expression of ion channels on target cell membranes. In addition, their indiscriminate biodistribution raises severe biosafety concerns. Nanotechnology offers a promising solution to these challenges.

### 3.1 Metallic nanomedicines promote DC activation

As the primary professional APCs, DCs play a central role in initiating antigen-specific antitumor immunity^157^. This process begins with the uptake of tumor antigens, which DCs then process into short peptide sequences. These peptides are then presented on the surface of DCs *via* major histocompatibility complex (MHC) molecules, facilitating the recognition and activation of T cells^6^^,^^158^. The immune functions of DCs are influenced by the integration of environmental signals through cytokines receptors and various PRRs^159^. However, these signaling pathways are often quiescent or impaired within the immunosuppressive TME^160^. Recent studies suggest that metal ions can act as immunomodulators by enhancing the sensing capabilities of PRRs such as the cyclic GMP–AMP synthase (cGAS)-STING signaling pathway, NLRP3 inflammasome and NF-κB signaling pathways^149^. This augmentation results in heightened maturation and activation of DCs, thereby reinforcing their effectiveness against cancer.

#### 3.1.1 Activation of cGAS–STING pathway

The cGAS–STING axis plays a pivotal role in immune detection and defense against pathogens and cancer^161^^,^^162^. Mechanistically, cGAS recognizes aberrant cytosolic double-stranded DNA (dsDNA) as a danger signal, triggering the production of cyclic GMP–AMP (cGAMP) as a second messenger to activate STING^163^. STING then initiates a downstream signaling cascade involving the phosphorylation of TANK-binding kinase 1 (TBK1) and interferon regulatory factor 3 (IRF3), leading to the induction of type I interferons (IFN-I) and other inflammatory cytokines to mount robust innate immune responses^164^. Du et al.^21^ discovered that DNA binding to cGAS triggered the formation of liquid-like droplets in which cGAS became activated. Free Zn^2+^ enhanced cGAS enzymatic activity by positively charging the N terminus of cGAS, which stabilized the cGAS–DNA phase separation. Conversely, depletion of intracellular Zn^2+^ by chelators inhibited cGAS–STING signaling.

**Figure 4:**
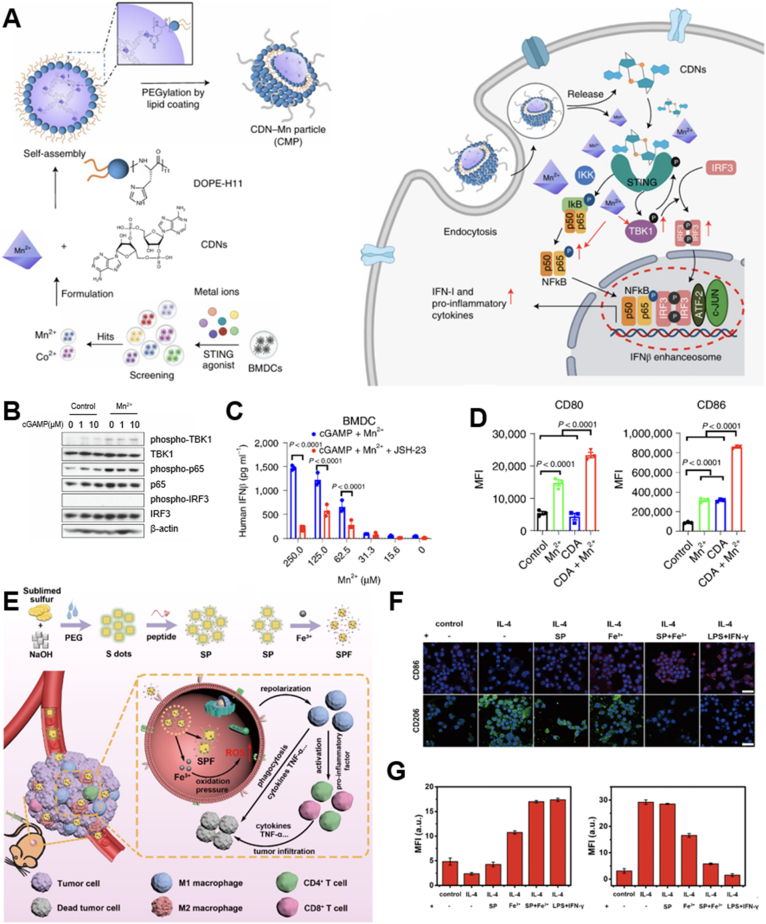
Representative examples of metallic nanomedicines modulating immune cells. (A) Preparation of a CDN-Mn coordination nanoparticle (CMP) and its molecular mechanism in boosting cGAS‒STING signaling. (B) STING activation in BMDCs after cGAMP treatment with or without Mn^2+^ supplementation. (C) IFN*β* production from BMDCs after cGAMP treatment with or without Mn^2+^ for 6 h. (D) Flow cytometric analysis of BMDC after different STING agonist treatment. Reprinted with the permission from Ref.^165^. Copyright © 2021, Springer Nature. (E) Preparation of an iron nanotrap to generate intracellular oxidation pressure for macrophage repolarization. (F) Immunofluorescent staining and (G) mean fluorescence intensity of CD86 (M1 biomarker) and CD206 (M2 biomarker) of RAW264.7 cells after indicated treatments. Scale bars: 20 μm. Reprinted with the permission from Ref. 196. Copyright © 2021, American Chemical Society.

Mn^2+^ was the first metal ion demonstrated to stimulate the cGAS–STING pathway, although the underlying mechanism remained controversial. Wang et al.^23^ found that Mn^2+^ not only sensitized cGAS to dsDNA, but also enhanced the binding capacity of cGAMP to STING. In contrast, Sun et al.^165^ utilized a thermal shift assay to conclude Mn^2+^ did not directly augment the binding between cGAMP and STING but instead induced the phosphorylation of TBK1 and NF-*κ*B (Fig. 4A–D). The authors further combined Mn^2+^ and cyclic dinucleotide in self-assembled coordination nanoparticles (CMP) to amply STING signaling, yielding a 12 to 77-fold increase in efficacy across multiple human STING haplotypes. The enhanced STING signaling by CMP substantially upregulated costimulatory markers CD80 and CD86 on DCs compared to the effects of single agonist of cGAMP or soluble Mn^2+^.

Inspired by these findings, a series of other Mn-containing nanoparticles have been developed as immune adjuvants for cancer treatment. For example, Jia et al.^166^ prepared hydrogel adjuvants (MnP^gel^) with a uniformly organized nanoscale microstructure by employing self-assembling peptides incorporated with Mn. This nanogel exhibited a sustained release profile of Mn^2+^, enhancing the phosphorylation of STING, TBK1, and IRF3 for DC maturation and activation compared to MnCl_2_. Furthermore, MnP^gel^ facilitated the formation of germinal centers, enhancing antibody production to levels comparable to conventional aluminum adjuvants. This suggests significant potential for its application in tumor vaccines. Similarly, Gao et al.^167^ developed a tumor nanovaccine (G5-pBA/OVA@Mn) by using benzoic acid (BA)-modified polyamidoamine dendrimer derivatives as carriers and combining Mn^2+^ with the protein ovalbumin (OVA). Upon entering the acidic endosomal compartment, the lowered pH diminished the coordination between Mn^2+^ and its ligands, promoting endosomal escape and facilitating the cytoplasmic delivery of both OVA and Mn^2+^. This process enhanced subsequent antigen cross-presentation through the cGAS–STING pathway. Flow cytometric analysis demonstrated that the G5-pBA/OVA@Mn formulation significantly increased the expression of co-stimulatory markers CD80 and CD86, with levels doubling compared to the OVA-only group. Additionally, cytokine secretion and CD8^+^ T cell activation were markedly higher in the G5-pBA/OVA@Mn group.

Bimetallic MOF nanoparticles combining Zn^2+^ and Mn^2+^ have also been designed to boost cGAS–STING pathway stimulation^168^. Specifically, Zn^2+^ enhanced H_2_O_2_ generation by inhibiting the mitochondrial electron transport chain, while Mn^2+^ converted accumulated endogenous H_2_O_2_ into toxic hydroxyl radicals. As a result, the bimetallic nanoparticles sustainably damaged mitochondria for mtDNA release for enhanced cGAS–STING activation than single ones. *In vitro* results confirmed that these bimetallic nanoparticles significantly upregulated the expression of CD40, CD80, and CD86 and the secretion of IFN-*β* compared to zinc nanoparticles alone.

In addition to Mn^2+^ and Zn^2+^, other metal ions such as Co^2+^ and Ln^3+^ have also been reported to activate the STING pathway^165^^,^^169^. For example, Luo et al.^170^ found all sixteen lanthanide ions exhibited STING activity. Next, the authors developed a series of Ln^3+^-based cGAMP-coordinated nanoparticles to amplify the immune effects, and europium-based nanoparticles (Eu-GAMP-NPs) were randomly selected from the nanoparticle families as an example to examine the immunological functions. The nanoparticles greatly enhanced the maturation and antigen presentation of DCs compared to single cGAMP. Eu-GAMP-NPs also augmented both humoral and cellular immune responses, accompanied by the secretion of multiple proinflammatory cytokines associated with the STING pathway. Recent studies have revealed that STING signaling can be spread from host cells to bystanders through the intercellular transfer of DNA or cGAMP^171^. It was therefore proposed that chemotherapeutics may stimulate STING functions when acting on tumor cells. Guo et al.^172^ encapsulated Pt nanoparticles and photosensitizers within hollow mesoporous organosilica nanoparticles. These nanoparticles were internalized by tumor cells, inducing the release of mtDNA and nuclear DNA, which then transferred to DCs to initiate cGAS–STING signaling. In another study, Ling et al.^173^ developed Pt-based photoactivators that catalyzed cGAS to produce cGAMP upon sensing damaged mitochondrial or nuclear DNA under laser irradiation. The cGAMP molecules released from dying tumor cells subsequently activated bystander DCs for immune stimulation.

#### 3.1.2 Activation of NLRP3 inflammasome

Aluminum salts (alum) are widely used as immune adjuvants in vaccine design by engaging the NLRP3 inflammasome and stimulating IL-1*β* production. However, alum commonly serves as a Th2 stimulator of the immune system and primarily enhances the antibody-mediated reaction, but with limited influence on the cytotoxic T cell-mediated response, which is considered the main mechanism of anticancer immune effects^174^. Sun et al.^175^ reported that, compared to conventional alum adjuvant, aluminum oxide nanoparticles (nano-alum) significantly augmented the anticancer effects of an immunotherapy based on tumor cell vaccines. However, the specific mechanism underlying this enhanced effect was not revealed in this study. To address, Sun et al.^176^ investigated the physicochemical properties of Al-based nanoparticles and their effects on immune responses. Specifically, they synthesized a series of aluminum oxyhydroxide (AlOOH) nanorods to determine whether controlling the shape and crystalline properties of the materials could provide insights into NLRP3 inflammasome activation and link the cellular response to the adjuvant activity of the material. It was demonstrated that crystallinity and surface hydroxyl group display of AlOOH nanoparticles significantly influenced the activation of the NLRP3 inflammasome in human THP-1 myeloid cells or murine bone marrow-derived dendritic cells (BMDCs). This activation led to elevated expression of MHC-II and multiple co-stimulatory molecules such as CD80, CD86, and CD40. Moreover, these *in vitro* effects were associated with enhanced immunopotentiation in a murine OVA immunization model, demonstrating the potential in cancer immunotherapy. These findings suggest that engineered Al-based adjuvants, combined with DC property–activity analysis, could be used to design more potent and tailored Al-based nanoadjuvants for improved immune responses.

Recent studies have also highlighted the role of electrophysiological responses in regulating DC function, with the concentrations of K^+^ and Ca^2+^ in the DC cytoplasm being closely linked to DC maturation. Based on this insight, Tan et al.^154^ synthesized various nanostructures, including Ph-Mg, Ph-Fe, and Ph-Zn, through ligand-driven self-assembly of Mg^2+^, Fe^2+^, and Zn^2+^ ions with l-phenylalanine. Fluorescence intensity and flow cytometric analysis revealed that the Ph-Zn significantly enhanced cellular uptake by DCs and activated them by modulating their electrophysiological properties. Mechanistically, the Zn^2+^-phenylalanine dimer altered the conformational structure of the K^+^ channel Kv1.3, leading to an influx of Ca^2+^ and the subsequent activation of the NLRP3 inflammasome and NF-*κ*B signaling pathways. This activation triggered the autocleavage of pro-caspase-1 in DCs, followed by the maturation of pro-IL-1*β* into its active cytokine form. Notably, Ph-Zn nanosheets induced the highest level of CD8^+^ T cell activation compared to other nanocomplexes containing Fe^2+^, Mg^2+^, Mn^2+^, Co^2+^, Cu^2+^, or Al^3+^. They also showed the best antitumor capacity when combined with PD-1 checkpoint blockade therapy.

#### 3.1.3 Other mechanisms

Apart from facilitating the STING pathway and NLRP3 inflammasome, metal-based nanoparticles also promoted DC maturation through regulating Ca^2+^ overload. Cao et al.^177^ synthesized calcium hydroxide nanoparticles coated with a layer of silica to enhance stability and conjugated them with anti-CD205 antibodies for targeted delivery to DCs. These calcium nanoparticles (AnCHNPs) promoted the expression of CD205 on the surface of DCs, efficiently entered the DCs and released Ca^2+^ in a regulated manner. The elevated cytoplasmic Ca^2+^ concentration activated both NFAT and NF-*κ*B pathways and promoted the expression of costimulatory molecules, antigen-presenting molecules, and pro-inflammatory cytokines. Transcriptome sequencing results further confirmed that AnCHNPs significantly elevated the expression of chemokines (*e*.*g*., *Cxcl1*, *Ccl5*, *Cxcl2*) and cytokines (*e*.*g*., *Il-1β*, *Il-12*, *and Il-6*), further enhancing DC-mediated antitumor immune responses. In contrast, CaCl_2_ as a control group failed to increase intracellular Ca^2+^ content and showed minimal effect in antitumor immune responses.

While metal-based nanoparticles have been extensively studied for their potential to boost DC activation, several mechanisms and challenges remain to be addressed. For example, Zn^2+^ plays a dual role in both inducing toxicity and activating innate immunity^178^. Further investigation is necessary to develop strategies that protect DCs from various forms of cell death^164^. Another possibility is that DCs may be more tolerant to Zn^2+^-induced cell mortality than tumor cells, though the underlying mechanism has yet to be elucidated. It is also worthwhile to note that the optimal concentration of Zn^2+^ for eliciting phase separation effect is reported to be between 160 and 625 μmol/L, at which point cGAS and DNA molecules are mobile and dynamically rearranged within the condensate with high bioactivity^21^. However, if the ion concentration surpasses this optimal range, the liquid structures may transform into a gelatinous state, constraining downstream signaling cascades^179^. This transformation may serve as an endogenous negative feedback mechanism to prevent overactivation of the innate immune response. Similarly, a recent study found that high concentration of Mn^2+^ (>5 μmol/L) inhibited the phosphorylation of STING and its downstream TBK1, ultimately suppressing rather than activating the immune system^179^. It's also important to recognize that both cGAS and STING proteins are widely expressed in normal tissue cells. Future studies should focus on mitigating the risk of cytokine storms, which can lead to autoimmune diseases due to excessive immune system activation^164^. In addition, at this stage, most studies focus on exploring the promotion of DC maturation by metal nanomaterials through the activation of cGAS–STING, while other pathways or mechanisms are lacking and need further investigation. The application of nanomedicines incorporating these metal ions in relation to DCs remains largely unexplored thus far, presenting new opportunities for cancer therapeutic interventions.

### 3.2 Metallic nanomedicines promote macrophage polarization

Tumor-associated macrophages (TAMs) are the predominant immune cell population within the TME^180^. Apart from their role as professional phagocytes that clear tumor cells, TAMs secrete various inflammatory factors shaping the immune landscape and also function as APCs to initiate adaptive immunity^181^. TAMs are typically classified into two main phenotypes: pro-inflammatory M1 and anti-inflammatory M2^182^. Unfortunately, a significant portion of TAMs display characteristics akin to the M2 phenotype, which not only impairs their ability to mount effective antitumor responses but also fosters tumor progression, metastasis, and resistance to treatment^183^. TAMs are highly plastic cells influenced by a range of environmental signals, indicating that shifting their polarization towards the M1 phenotype could be a promising strategy for immunotherapy. This section reviews the strategies for metallic nanomedicines promoting macrophage polarization through different mechanisms.

#### 3.2.1 Activation of cGAS–STING pathway

cGAS–STING pathway has also been implicated in reprogramming TAMs to immunopermissive state. Tian et al.^184^ developed an amphiphilic metal-phenolic nanomaterial for targeted delivery to mitochondria by coordinating Mn^2+^ with an amphiphilic phenolic polymer. This nanomaterial combines mitochondria-targeted photodynamic therapy (PDT)-induced oxidative stress with furan-mediated DNA cross-linking, leading to excessive mtDNA damage. The resulting release of mtDNA fragments activates the cGAS–STING signaling pathway in TAMs. Additionally, Mn^2+^ synergistically repolarized M2-like TAMs toward an M1-like phenotype and enhanced T cell-mediated antitumor immunity. Flow cytometric data demonstrated that the combination of Mn^2+^ and phenolic polymer significantly increased the proportion of M1-type macrophages and the secretion of M1 characteristic cytokines such as TNF-*α* and IFN-*β* compared to Mn^2+^ or phenolic polymer alone. Western blot analysis further confirmed that key STING pathway indicators, including pTBK1 and pIRF3, were upregulated in response to this synergistic effect. The nanomaterial demonstrated the most effective tumor inhibition, achieving approximately 85% growth suppression by Day 14 compared to the PBS control group. In contrast, Mn^2+^ or the phenolic polymer alone exhibited only marginal therapeutic effects. Notably, the use of the STING inhibitor C178 completely abrogated the immune response, providing mechanistic evidence for the dependence on the cGAS–STING pathway.

To enhance macrophage targeting of metallic nanodrugs, Li et al.^185^ developed a mammalian porphyrin-like core-shell nanostructure (Rm@PP-GA) designed for CD163 targeting. The shell mimicked the membrane structure of red blood cells, with Rm serving as a carrier to prolong the circulation time of the drug *in vivo*. This design allowed the nanostructure to target CD163 receptors on tumor cells. Manganese porphyrin within the structure acted as a photo-STING agonist, generating damaged mtDNA under 638 nm light irradiation. This, in turn, synergistically activated the macrophage cGAS–STING pathway in conjunction with Mn^2+^. Compared to manganese porphyrin alone, Rm@PP-GA significantly enhanced the phosphorylation of STING-related proteins, likely due to increased cellular uptake that facilitates greater Mn^2+^ delivery for more effective STING pathway activation. Flow cytometric data showed that the expression of CD86 in macrophages was markedly elevated following treatment. Exposure to light conditions further amplified M1 macrophage polarization, with CD86 levels 1.7 times higher than in untreated cells. Additionally, cytokines secreted by M1-like macrophages, such as IL-6 and TNF-*α*, increased 27.6- and 26.1-fold, respectively. These results confirm that Rm@PP-GA significantly promoted macrophage polarization and achieved tumor eradication through activation of the cGAS–STING pathway.

#### 3.2.2 Activation of TLR

TLR refers to a class of receptors that are integral to the innate immune system, playing a crucial role in pathogen recognition and the initiation of immune responses. Taking TLR4 as an example, it is a key pattern recognition receptor primarily expressed in macrophages, responsible for detecting lipopolysaccharide (LPS) and bacterial endotoxins^186^. Activation of TLR4 triggers the production of cytokines such as *Il-1β*, *Il-6*, and *Tnf-α,* which contribute to the polarization of macrophages towards the M1 phenotype^187^. Recent studies have shown that trace amounts of Mn^2+^ can directly activate TLR4 by mimicking pathogen-associated molecular patterns. Building on this discovery, Huang et al.^188^ developed Mn^2+^-encapsulated albumin nanocomplexes (Mn@BSA) to stimulate pro-inflammatory responses in TAMs for cancer immunotherapy. Mn@BSA activated TLR4 and its downstream signaling pathways, including NF-*κ*B and activator protein 1, alongside the nuclear translocation of interferon IRF7 for IFN-I production. Consequently, compared to Mn^2+^ alone, Mn@BSA nanocomplexes significantly upgraded the mRNA levels of TLR4-related intermediators such as MyD88, TRIF and IRF7 as well as the expression of M1 polarization markers on macrophages, such as CD64, CD80, and CD86. When assembled into nanowire structures, the Mn@BSA complexes further enhanced their immunostimulatory effects by modulating phagocytosis, thereby prolonging the immune activation of macrophages. Notably, Mn@BSA demonstrated improved biosafety compared to the clinically used TLR4 agonist LPS, highlighting its potential for advancing cancer immunotherapy.

In addition to Mn-based nanoparticles, Ag-based nanocomplexes (AgNCs) have also been developed to promote the repolarization of TAMs through the TLR4 signaling pathway characterized by the upregulated expression of IL-6 and inducible nitric oxide synthase (iNOS)^189^^,^^190^. As a result, the activated macrophages secrete a range of inflammatory factors into the TME, triggering and sustaining antitumor immune responses. In murine studies, macrophages engineered with AgNCs exhibited significant targeting ability toward tumor sites and effectively inhibited lung metastasis in breast cancer. Additionally, the intrinsic fluorescence of AgNCs allows for *in vivo* tracking of macrophages, positioning them as an ideal theragnostic platform for cancer treatment.

#### 3.2.3 Regulation of iron metabolism

Recent studies have highlighted distinct differences in iron metabolism between M1 and M2 macrophages^191^^,^^192^. Generally, M1 macrophages express relatively high levels of ferritin, an iron storage protein, and low levels of ferroportin, an iron export channel, compared to M2 macrophages^193^. This suggests that intracellular iron overload in macrophages may serve as effective inducers of this polarization switch. Ferumoxytol, a Food and Drug Administration (FDA)-approved iron oxide nanoparticle used for treating anemia, has been found to increase ROS levels *via* the Fenton reaction in macrophages^194^. This promoted M1 polarization and upregulated key markers such as TNF-*α* and CD86, which in turn inhibited breast cancer progression and liver cancer metastasis. Other iron-based nanoparticles like magnetite, magnetohematite, and mixed ferrite have also demonstrated similar ability to promote macrophage repolarization following analogous mechanisms^195^. Sang et al.^196^ utilized a different strategy to achieve iron overload by employing sulfur quantum dots (S-QD) as nano-traps for free iron ions (Fig. 4E–G). S-QD had a high affinity for iron ions, allowing them to adsorb substantial amounts of endogenous iron in the TME and deliver them specifically to TAMs. Within TAMs, the acidic lysosomal conditions and intracellular H_2_O_2_ triggered the release of iron from the nano-trap, generating ROS that reprogramed TAMs towards M1-like phenotypes. This study serves as an example of harnessing endogenous irons to mitigate the potential toxicity associated with introducing exogenous metal ions.

Interestingly, macrophages differ in their sensitivity to ferroptosis, with M1 macrophages showing significantly greater resistance to intracellular iron accumulation compared to their M2 counterparts^191^. This resistance is likely due to the detoxifying effect of overexpressed iNOS in M1 macrophages. In other words, ferroptosis may selectively eliminate immunosuppressive M2 macrophages without harming the immunosupportive M1 macrophages^197^. Chen et al.^198^ constructed a bionic ferroptosis inducer by co-loading dihydroartemisinin (DHA) and Fe^3+^ into hollow mesoporous silica nanoparticles, which were further modified with bacterial outer membrane vesicles. This nanoformulation rapidly dissociated in the acidic TME, where the released Fe^3+^ was reduced to Fe^2+^ by consuming intratumoral GSH. Fe^2+^ and DHA coordinated to trigger the Fenton reaction, generating ROS that induced ferroptosis in both tumor cells and TAMs. Notably, the ferroptosis-sensitive M2 macrophages either succumbed to ferroptosis or gradually transitioned into ferroptosis-resistant M1 macrophages under Fe^2+^ stress. As a result, significantly induced M1 polarization of TAMs and downregulated the expression of M2-associated cytokines compared to the DHA-only group. This transformation promoted the reprogramming of the immunosuppressive TME and ultimately enhanced the efficacy of ferroptosis-induced cancer immunotherapy.

Recent findings indicate that the regulation of macrophage polarization by Fe-based nanoparticles could be influenced by several factors, including size, surface charge, morphology, and other physiochemical properties^199^. For instance, Cheng et al.^200^ found that smaller Fe_3_O_4_ nanoparticles (4 nm) are more effective in inducing the polarization of M1 macrophages, likely due to their higher cellular uptake rates and greater intracellular accumulation. Furthermore, external factors such as magnetic fields, temperature, and laser irradiation can exert impact on the macrophage polarization process, opening up possibilities for designing multifunctional Fe-based nanoplatforms.

#### 3.2.4 Other mechanisms

Precious metals such as Au, Ag, and Pt were also found with strong capability to repolarize macrophages due to their exceptional catalytic activity towards H_2_O_2_^201^. This activity regulates intracellular ROS and reactive nitrogen species production, thereby triggering inflammatory pathways including TLR, MAPKs, and NF-*κ*B, all of which contribute to macrophage polarization. Moreover, increasing evidence indicates that intracellular Ca^2+^ plays a significant role in macrophage activation and phenotypic transformation^202^. Elevated levels of intracellular Ca^2+^ can promote the phosphorylation of IKK*β*, thereby activating NF-*κ*B and MAPK signaling pathways to support TAM polarization^203^. Meanwhile, increased Ca^2+^ concentrations can activate NLRP3 inflammasomes by elevating ROS levels in mitochondria, leading to the release of pro-inflammatory cytokines like IL-1*β*. Building on this understanding, An et al.^204^ proposed a multifunctional Ca^2+^-based platform (CaNP@cAD-PEG), integrating ultra-high pH-sensitive calcium peroxide nanoparticles (CaNPs) with circular aptamer-DNAzyme conjugates (cAD), to eradicate cancer through multiple synergistic mechanisms termed “calcium interference”. In detail, CaNP and cAD disassembled in the acidic TME to act independently in different cells. CaNPs taken up by TAMs significantly activated NLRP3 inflammasomes and reset TAMs towards the M1 phenotypes. Concurrently, CaNPs internalized by tumor cells provided Ca^2+^ as cofactors to facilitate DNA enzyme-mediated depletion of PD-L1, thereby synergistically amplifying antitumor immune responses.

It is worthwhile to note that, recent findings also suggested that Ca^2+^ depletion within the ER can polarize TAMs toward an antitumor phenotype, enhancing their ability to present antigens to T cells and activate the adaptive immune response^205^. Additionally, Ca^2+^ depletion in tumor cells can disrupt intercellular junctions, breaking down the physical barrier of solid tumors and facilitating immune cell infiltration. In another study, it was demonstrated that elevated intracellular Ca^2+^ levels in tumor cells triggered the release of exosomes containing PD-L1, which suppressed effector T cell function and contributed to tumor progression in mouse models^206^. Above studies suggest the complex role of Ca^2+^ in onco-immunity, while its reliance on dose, cell type, and tumor stage requires further investigation before clinical translation.

Taken together, the polarization of TAMs by metallic nanomedicine involves a range of mechanisms that often interact, if not overlap. Even within the scenario of Mn^2+^-based nanoparticles, they mediate the activation of cGAS–STING axis, NLRP3 inflammatory vesicles, TLR signaling pathway, and others, all of which have been shown to contribute to TAM repolarization^188^^,^^207^. It would be interesting and valuable to explore which of these mechanisms is most effective for macrophage polarization and how they might work synergistically to combat cancer^208^. Additionally, given the presence of multiple cell types within tumor tissues, how to differentiate the specific effects of macrophage polarization from those of DC activation or direct RCD effects on tumor cells? Single-cell analysis of the TME or cell-targeting delivery approaches may provide insights into these questions and ultimately enhance oncotherapy^209^.

### 3.3 Metallic nanomedicines modulate other immune cells

Myeloid-derived suppressor cells (MDSCs) are important motivators to drive tumorigenesis and promote tumor immune escape^210^, ^211^, ^212^. They can also inhibit the cytotoxic activities of effector T cells and NK cells through various mechanisms including expressing immune checkpoint inhibitors, consuming essential amino acids, secreting immunosuppressive cytokines, and creating hypoxic environment^213^. Zhu et al.^214^ engineered H6-coupled gold nanoparticles to directly target and modulate MDSCs. The resulting nanosystem effectively scavenged ROS and inhibited NLRP3 inflammasomes activation through blocking NLRP3–NEK7 interaction, thereby reducing IL-1*β* production and diminishing the MDSC population in the TME. Consequently, these nanoparticles reversed the immunosuppressive functions of MDSCs.

NK cells are innate lymphocytes known for their cytotoxicity against tumor cells without relying on MHC molecules^215^. They also secrete various cytokines that modulate the activities of other immune cells like DCs, TAMs, and T cells^151^. Multiple clinical trials are underway to explore the combination of chemotherapy with NK cell transfer as a strategy to combat cancer. However, the cytotoxic function of NK cells is often suppressed in the TME. Chen et al.^216^ synthesized a ruthenium polypyridine complex (Ru-POP) aimed at potentiating adoptive NK cells transfer therapy against breast cancer. Ru-POP activated multiple apoptosis-related receptors like TNF-R1, DR5, Fas on tumor cells, maximizing the interplay between tumor cells and NK cells *via* up-regulating natural killer group 2 member D to facilitate tumor clearance, although the precise molecular mechanism remained unclear.

CD8^+^ T cells have become a central focus in orchestrating the adaptive immune system to combat cancer^217^. However, the immunosuppressive TME restricts the infiltration of these T cells, contributing to the low response rate of immune checkpoint inhibitors and chimeric antigen receptor T-cell (CAR-T) therapies^218^. To tackle this challenge, Zou et al.^219^ developed chromium (Cr) nanoparticles embedded in biodegradable polydopamine shells to enhance the migration and infiltration of CAR-T cells. These nanocomposites (Cr@PD) released Cr^3+^ in the acidic TME, thereby upregulating the expression of C–X–C motif chemokine ligand 13 (*Cxcl13*) and CC-chemokine ligand 3 (*Ccl3*), which promoted the formation of tertiary lymphoid structures (TLSs) within the tumor tissues. Subsequent administered CAR-T cells efficiently infiltrated and accumulated in these TLSs, leading to heightened secretion of cytotoxic cytokines including IL-2, IFN-*γ*, and TNF-*α*. As a result, Cr@PD significantly empowered CAR-T therapy, inhibiting tumor growth and prolonging animal survival.

To date, only limited research has explored the use of metallic nanoparticles to directly modulate T cells function, largely because they are typically non-phagocytic. Accumulating evidence highlights the direct influence of metal ions on T cell biology, impacting differentiation, proliferation, infiltration, and cytotoxic functions^220^^,^^221^. For example, elevated intracellular Ca^2+^ levels could prompt the dissociation of the CD3*ε*/*δ* cytoplasmic domain from the T cell membrane, exposing the immunoreceptor tyrosine-based activation motif (ITAM) residues on the TCR complex^18^. This exposure allows for electrostatically interaction with acidic phospholipids, facilitating ITAM phosphorylation, which is the initial step in antigen recognition by T cells. Consequently, Ca^2+^ enhances TCR's response to relatively weak antigenic stimulation signals, increasing the sensitivity of CD8^+^ T cells to foreign antigens^222^. Additionally, the Ca^2+^ influx during T cell activation causes plasma membrane depolarization, which stimulates an open state of Kv1.3. The subsequent efflux of K^+^ through Kv1.3 further repolarizes the cell membrane, maintaining the driving force for sustained Ca^2+^ entry, crucial for effector T cell activation and proliferation^223^. Extracellular K^+^ abundance has also been reported to impair T cell function by interfering with TCR-driven Akt-mTOR phosphorylation^224^. Therefore, strategies to chelate K^+^ could potentially enhance CD8^+^ T cell-mediated antitumor immunity. Interestingly, recent studies suggest that the excess K^+^ may preserve the stemness of a small subset of T cells through limited nutrient uptake and epigenetic modification^17^. These stem T cells exhibit self-renewal, expansion, and multipotency, contributing to the eradication of large metastatic tumors. This dual role of K^+^ underscores its complex impact on T cell activity in tumor immunology, necessitating comprehensive study before therapeutic application. As nanotechnology comes of age, more metal-based nanomedicine approaches are expected to advance T-cell based cancer immunotherapy, including innovations like CAR-T backpacks^219^. The integration of nanotechnology and immunology promises to deepen our understanding of the tumor immune contexture and ultimately contribute to cancer therapy.

In addition to directly activating immune cells, metallic nanomedicines can also enhance antitumor immune response by reversing the immunosuppressive TME, which is characterized by acidic pH, hypoxia, ROS overexpression, and others^225^. These features not only promote tumor cell growth and metastasis, but also impair the infiltration and activation of immune cells as well as cytotoxic functions. For example, TAMs typically accumulate in the hypoxic regions of solid tumors^226^. This hypoxic environment stimulates TAMs to produce higher levels of vascular endothelial growth factor, lymphangiogenesis, and multiple tissue remodeling-related factors, which facilitate cancer metastasis and invasion^227^. Thus, alleviating the local hypoxic environment within tumors has emerged as a viable strategy to shift macrophage polarization. Based on this idea, MnO_2_ nanoparticles were developed to catalyze the conversion of H_2_O_2_ into O_2_, effectively reducing tumor hypoxia and inhibiting the generation of M2-polarized macrophages^228^. Similarly, MnO_2_ nanozymes coated with tumor cell membranes (CM@Mn) were engineered to alleviate hypoxia in the TME^207^. The concurrent release of Mn^2+^ from these nanozymes further contributed to TAM repolarization *via* the cGAS–STING pathway, enhancing their antigen-presenting capacity and promoting T cell-mediated immune responses. In murine tumor models, CM@Mn not only inhibited the proliferation of primary and metastatic tumors but also demonstrated a long-term immune memory effect against tumor rechallenge.

The extracellular matrix (ECM) surrounding tumors poses a barrier to immune cells infiltration and influences the recognition and uptake of tumor antigens by DCs^229^. Recent studies pointed out that Zn^2+^ participation was required in the activation of matrix metalloproteinases (MMPs), a family of endogenous proteolytic enzymes mediating the degradation of collagen components in the ECM^230^. Building on this, Ding et al.^231^ engineered multifunctional Zn-based MOF nanoparticles capable of site-directed release of Zn^2+^ in the acidic TME. They promoted ECM degradation through upregulating MMP-2's activity, thus significantly enhancing tumor infiltration and killing CD8^+^ T cells. This finding provides novel insights into utilizing metallic nanomedicine approaches to overcome the physical barriers surrounding tumor tissues, thereby alleviating the immunosuppressive TME.

Despite their potential, many metallic nanoparticles can exhibit toxicity toward immune cells under certain conditions. Prolonged exposure to TiO_2_ nanoparticles, for example, has been shown to significantly reduce the number of NK cells^232^. Factors such as concentration, shape, and size of the nanoparticles play critical roles in determining their efficacy-safety profiles^233^^,^^234^. Gold nanoparticles at a concentration of 0.25 ppm can effectively enhance the expression of pro-inflammatory cytokines like IL-1*β*, IL-6, and TNF-*α*^235^. However, when the dosage exceeds 25 ppm, the same nanoparticles can diminish the proliferative activity of lymphocytes. Additionally, rod-shaped gold nanoparticles are more likely to suppress immune responses, which can disrupt cell membrane integrity^236^. Al_2_O_3_ nanoparticles have been shown to alter cytokine levels in normal organs and blood circulation, potentially leading to immune-related organ dysfunction and cytokine storms^237^. Moreover, immunomodulatory responses to metal substances can vary widely among species and may be further influenced by factors such as age, sex, genetics, and metabolic state^238^. Therefore, experimental data from animal models do not always translate effectively into clinical settings. A comprehensive understanding of the potential risks is essential for developing safe metallic nanoparticles.

## 4 Metallic nanoparticles as carriers for drug delivery

Beyond their direct immunomodulatory function, metal materials also hold promise as potential carriers for the delivery of therapeutic agents^239^. These agents can either be adsorbed onto the dense surface or encapsulated within the internal cavities of metal carriers^240^. In some cases, the agents themselves can function as integral components of nanosystems, leading to significantly higher loading capacities compared to traditional organic delivery vehicles. This is particularly evident with hydrophilic biomolecules, such as proteins, peptides, amino acids, and nucleotides, which are rich in functional groups (*e.g.*, amino, carboxyl, and heterocyclic oxygen groups) that readily form coordination bonds with metal ions^241^. Metal-based carriers also increase the water solubility and dissolution rates of hydrophobic agents while improving their physiochemical stability^242^. The dense surface and tailored structure of metallic nanoparticles allow straightforward chemical modification with functional moieties to optimize ADME processes such as extending circulation half-life, optimizing biodistribution profile, and introducing responsiveness to pathological cues^243^^,^^244^. Moreover, metal elements possess outstanding optical and thermal properties that can be leveraged for controlling drug release as well as monitoring pharmacokinetics of agents for theragnostic purposes^245^. Below, we listed major therapeutic advantages of metallic materials as drug carriers compared to their non-metallic counterparts.

### 4.1 High drug loading and improved stability

The substantial surface area of metal nanoparticles provides more interaction sites for the adsorption or encapsulation of drug molecules compared to conventional organic delivery carriers, which is a key factor contributing to their high drug-loading capacity^239^. Drug molecules can be encapsulated within metal nanoparticles through non-covalent interactions, primarily involving electrostatic and hydrophobic forces^246^. For example, Zhang et al.^247^ incorporated STING agonist SR717 and indoleamine 2,3-dioxygenase 1 (IDO1) inhibitor curcumin into Zn^2+^-based nanoparticles, achieving loading efficiencies of 17.2% for SR717 and 78.6% for curcumin. These metal-based nanoparticles significantly enhanced tumor accumulation *in vivo* compared to free drug molecules, whose clinical efficacy is often hindered by non-specific biodistribution. Consequently, the nanoparticles not only effectively activated the STING pathway but also inhibited the IDO1 immune checkpoint within the TME, thereby promoting IFN-*β* secretion, DC maturation, and effector T cell infiltration to combat cancer.

Drug molecules, particularly biomolecules, can also be covalently attached to metal-based nanoparticles to enhance their biostability^248^. For example, ganoderma lucidum polysaccharides (GLP), known for their potent immunomodulatory effects on DC maturation, face limitations in antitumor efficacy due to rapid clearance *in vivo*^249^. Zhang et al.^250^ developed nanocomposites by conjugating the sulfhydryl groups of GLP to gold nanoparticles, effectively prolonging its circulation half-life. The resulting GLP-Au nanocomposites exhibited stronger DC activation compared to free GLP, evident by the increase of costimulatory factor expression and pro-inflammatory cytokine transcription. Similarly, Luo et al.^251^ conjugated ovalbumin (OVA) antigen to ultra-small Fe_3_O_4_ nanoparticles *via* amidation, achieving a 97% loading capacity for OVA. The Fe_3_O_4_-OVA nanovaccine not only protected antigens from degradation in circulation but also effectively stimulated DC maturation and induced robust T cell responses against both subcutaneous and metastatic tumors, outperforming soluble tumor antigens.

Some immunotherapeutic agents, particularly those with multivalent functional groups, can serve as building blocks within the nanosystem^252^. Clinical trials of the cytosine-phosphate-guanine oligodeoxynucleotides (CpG-ODNs), an immunostimulatory molecule tested in clinical trials have progressed to phase III^253^. However, CpG-ODNs face challenges such as limited stability in serum, often requiring repeated high-dose administrations, which can result in toxic side effects and reduced efficacy. To overcome this issue, Chen et al.^254^ coordinated CpG-ODNs with ZrO clusters through the formation of Zr-O-P bonds to construct a MOF structure. This approach achieved a significantly higher CpG-ODN loading ratio (8.3%) compared to other delivery systems, such as gold nanoparticles, poly(l-lysine)-functionalized silica nanoparticles, or zeolitic imidazolate framework-8 (all less than 5.0%). The CpG-ODNs showed almost no release in the presence of bovine serum albumin, demonstrating excellent serum stability. Furthermore, the nanoparticles activated the TLR4 signaling pathway at an extremely low dose (70 ng/mL), effectively promoting DC maturation without systemic immune-related toxicity^255^.

Overall, metal nanoparticles offer versatile methods for delivering various types of therapeutic immunoagents with higher drug loading capacity and improved stability compared to conventional organic delivery carriers. Covalent interactions generally provide strong binding affinity between drug molecules and metal carriers, helping to prevent premature drug leakage and potentially reducing off-target biodistribution compared to non-covalent interactions^256^. However, the fabrication of such systems often requires stringent reaction conditions, which can make it challenging to preserve the drug's bioactivity during the process. Furthermore, the drug molecules must eventually detach to interact with their therapeutic targets, necessitating a delicate balance between preparation stability (off) and drug release (on)^257^. In this context, stimuli-responsive metallic nanosystems that remain stable in circulation but dissociate at desired sites in response to endogenous or exogenous triggers may represent an ideal approach. For example, Yu et al.^258^ coordinated double-stranded siRNA with the phosphate backbones in NaGdF4 nanoparticles. Under acidic endosomal conditions, protonation of phosphate groups reduced siRNA affinity to NaGdF4 nanoparticles, leading to siRNA dissociation. This effect allowed dissociated siRNA molecules to escape from endosomes *via* the proton sponge mechanism, thereby achieving efficient gene silencing of PD-L1 for enhanced cancer immunotherapy.

### 4.2 Tunable size and shape

Nanomedicines with a size range between 10 and 200 nm are prone to accumulating at solid tumor sites due to their ability to penetrate tumor blood vessels through inter-endothelial gaps, enhancing drug efficacy while reducing undesired exposure in healthy tissues^259^^,^^260^. This phenomenon is known as the enhanced permeability and retention (EPR) effect, or passive targeting^261^. Metallic nanoparticles, in particular, offer more tunability in size compared to organic nanoparticles, allowing for greater precision in control over their properties. Liu et al.^262^ compared the *in vivo* distribution of gold nanoparticles of varying sizes using surface-enhanced Raman spectroscopy and found that tumor accumulation followed the order of 34 nm > 147 nm > 108 nm > 60 nm. The authors hypothesized that the 34 nm nanoparticles exhibited more favorable diffusion constants and prolonged residence times, thereby enhancing their accumulation within the TME. In contrast, nanoparticles sized at 60 nm were primarily localized in the periphery of the tumor or adjacent to the blood vessels, thus being more likely to be washed away by the bloodstream. Cruz et al.^263^ further demonstrated that nanoparticles sized at 50 nm exhibited a fourfold higher cellular uptake compared to those of 20 nm size. However, further increases in nanoparticle size resulted in decreased cellular endocytosis efficiency, likely due to the cut-off size limitation (50 nm) of endothelial gaps in tumor vessels.

**Figure 5:**
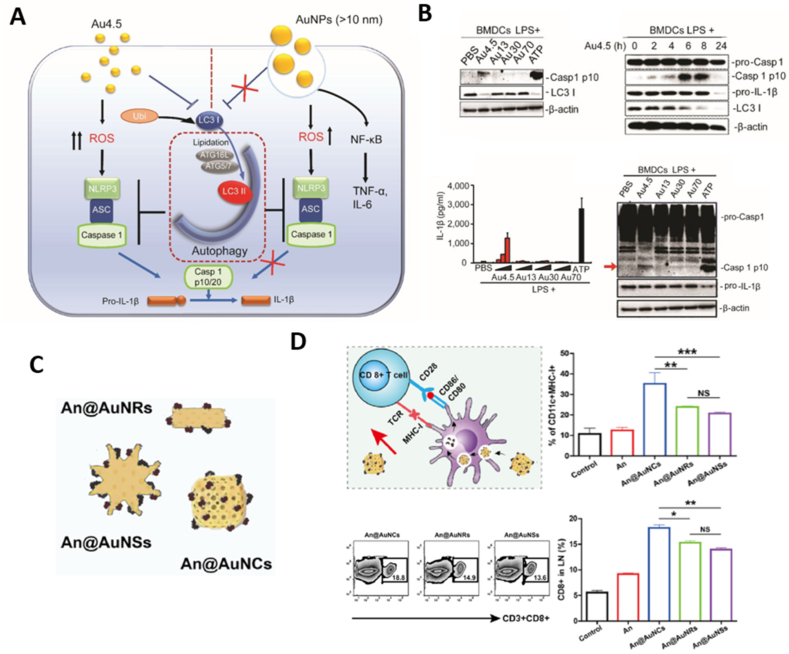
Representative examples of influencing immunomodulatory effects by varying particle size and shape of metal nanoparticles. (A, B) Schematic illustration and evaluation of the effects of gold nanoparticles of different sizes on mediating NLRP3 inflammasome activation. Reprinted with the permission from Ref. 265. Copyright © 2020, American Chemical Society. (C, D) Schematic illustration and evaluation of the effects of gold nanoparticles with different shapes on the presentation of peptide-MHC-I complexes. Reprinted with the permission from Ref. 267. Copyright © 2023, Elsevier B.V.

The size of metallic nanoparticles also determines their immunomodulatory effects. Zhou et al.^264^ compared the immunological effects of gold-based nanovaccines sized at 60 nm and 80 nm, both co-loaded with ovalbumin peptide as a model antigen and CpG oligonucleotide as an immune adjuvant. Interestingly, the 60 nm nanovaccines were more effective in enhancing antigen presentation, while the 80 nm nanovaccines induced higher levels of costimulatory molecules on DCs. A combination of these two nanovaccine sizes elicited the strongest antigen-specific CD8^+^ T cell responses against tumors. Zhu et al.^265^ reported smaller metallic nanoparticles were more efficient in blocking autophagy. Specifically, ultra-small gold nanoparticles (4.5 nm) effectively inhibited the degradation of the autophagy protein microtubule-associated protein light chain 3 (LC3) by activating the NLRP3 inflammasome (Fig. 5A and B). In contrast, larger gold nanoparticles (>10 nm) were more effective at triggering the NF-*κ*B signaling pathway. These findings provide valuable insight into controlling nanoparticle size in selectively modulating innate immune pathways to enhance immunotherapy.

The geometry of nanoparticles is another critical factor. Bhoge et al.^266^ observed that star-shaped gold nanoparticles tended to stay in the late endosome and lysosome regions, while rod-shaped gold nanoparticles were predominantly sequestered in the mitochondrial region. Therefore, when loading CpG, which targets TLR9 expressed on the inner surface of endosomes, the star-shaped gold nanoparticles demonstrated more effective inflammatory responses to boost DC activation. Similarly, Zhao et al.^267^ found that cage-shaped gold-based nanovaccines preferentially presented peptide-MHC-I complexes, leading to stronger CD8^+^ T cell immunity compared to rod and star-shaped nanovaccines (Fig. 5C and D). Yang et al.^268^ investigated how the structural properties of Mn-based nanoparticles influence their immunomodulatory effects. They synthesized a series of spherical and rod-shaped hollow MnO_2_ nanoparticles with varying diameters and aspect ratios. Their findings highlighted that microrod nanoparticles sized at 151 nm with an aspect ratio of 4.5 exhibited superior capabilities in stimulating the activation of diverse DC subsets and promoting infiltration of antigen-specific CD8^+^ T cells.

Overall, the ease of synthesis and tunable physical properties of metallic nanoparticles offer significant advantages in precisely controlling their *in vivo* fate and immunological effects, outperforming conventional organic nanoparticles. The size-dependent EPR effect has long been considered a dominant mechanism for the accumulation of nanoparticles in tumor tissues^269^. However, recent studies involving gold nanoparticles have challenged this view, suggesting that transcytosis through endothelial cells may be the primary route for tumor entry^270^^,^^271^. Beyond the mechanisms governing nanoparticle entry into solid tumors, the exit of nanoparticles is equally important as it also contributes to the overall tumor accumulation behavior^272^. However, comparatively little effort has been directed toward this field. Other factors such as charge, stiffness, elasticity, chemical composition may also influence metallic nanoparticles’ behavior and require further investigation.

### 4.3 Easy surface engineering

Due to the high surface area and abundant active chemical groups, metallic nanoparticles can be easily functionalized to deliver therapeutic agents with “active targeting” capabilities^273^. For example, conjugating trastuzumab, which recognizes HER2 receptors overexpressed in breast tumors, to gold nanoparticles using standard bioconjugate chemistry showed enhanced tumor accumulation and prolonged therapeutic effects^274^. Interestingly, better targeting efficiency was observed when nanoparticles were conjugated with exactly one trastuzumab molecule instead of two, underscoring the importance of precise surface decoration for optimal active targeting. Metallic nanocarriers can also modify drug behaviors at the cellular level. Ramucirumab is currently the sole FDA-approved targeted drug for treating advanced and metastatic gastric adenocarcinoma, but its effective dosage (8 mg/kg) often leads to associated toxicity^275^. Recent findings indicated that metallic nanoformulations with high density are more readily taken up by cells compared to their non-metallic counterparts^276^. In a study by Fan et al.^277^, ramucirumab was conjugated onto the surface of gold nanoparticles to enhance its cellular uptake efficiency. This approach facilitated robust activation of immune receptor and phagocytosis related pathways at a relatively safer dosage (1 mg/kg). In contrast, neither the antibody nor the gold nanoparticles alone exhibited antitumor effects.

On-demand drug delivery with organelle-level precision holds significant promise for improving therapeutic outcomes. PDT induces ROS through ER stress, while the clinically used photosensitizer indocyanine green (ICG) mainly localizes in the cytoplasm after cell internalization^278^. Such a paradoxical situation largely limits the occurrence of PDT-induced immunogenicity. To address this issue, Li et al.^132^ developed ER-targeting coordination nanosystems by conjugating pardaxin peptides onto the surface of ICG-encapsulated hollow gold nanospheres, inducing significant ER stress and ROS production. T-cell immunotherapy encounters significant obstacles in solid tumors, partly due to the insufficient synthesis and release of cytotoxic proteins stored in lysosomes, which cannot effectively penetrate tumor cell membranes to induce apoptosis. To address this, Zhao et al.^279^ developed Zn^2+^-based MOF nanoparticles pre-loaded with perforin and granzyme B, which were further coupled with a CD63 aptamer targeting the lysosomal compartment. In the acidic lysosomal environment, these nanoparticles disassociated, releasing perforin and granzyme B. Upon recognition of tumor cells by T cells, the lysosomal contents are discharged to the immunological synapse, enabling precise and efficient tumor cell killing.

In summary, surface functionalization of metallic nanoparticles provides significant advantages in delivering immunotherapeutic agents with precise targeting at the tissue, cell, and organelle levels^112^^,^^277^. However, this process inevitably introduces complexity in the synthesis and may result in the incorporation of non-degradable or toxic ligands^280^. Therefore, robust synthesis strategies and thorough biocompatibility examinations are essential early in the development process to ensure scalability and successful translation into clinical practice.

### 4.4 Combination immunotherapy

The inherent immunological properties of metal substances provide a versatile platform, especially when combined with their cargo, to overcome drug resistance and enhance therapeutic efficacy. For example, Fe_3_O_4_ nanovaccines were developed to co-deliver OVA antigens and CpG adjuvants to DCs^281^. The Fe_3_O_4_ nanoparticles not only facilitated cargos uptake by DCs through membrane fusion and endosome-mediated endocytosis, but also acted as immune adjuvants by generating intracellular ROS for DC maturation. In another study, Huang et al.^282^ engineered Mn–Zn bimetallic MOF nanoparticles encapsulating CRISPR plasmids to combine metabolic therapy and immunotherapy against cancer. In the design, the nanoparticles facilitated cellular uptake and intracellular transfection of the plasmids, leading to the downregulation of the methionine transporter protein SLC43A2 on the surface of tumor cells. This resulted in elevated methionine levels in the TME, thereby alleviating methionine competition pressure on CD8^+^ T cells. Furthermore, the released Mn^2+^ and Zn^2+^ effectively activated the cGAS–STING pathway, synergistically enhancing infiltration and proliferation of CD8^+^ T cells. Consequently, this nanoplatform demonstrated robust immunotherapeutic efficacy in preclinical cancer models.

**Figure 6:**
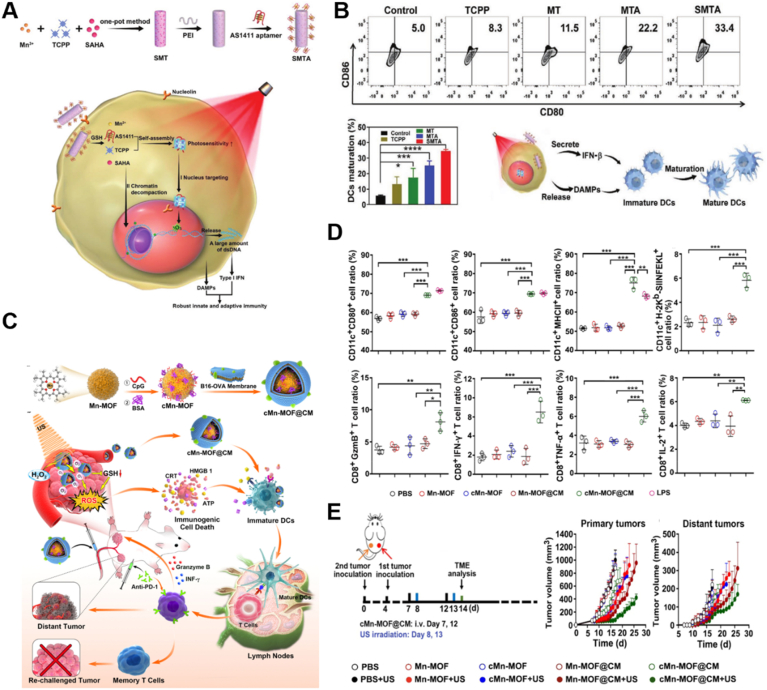
Representative examples of metallic delivery systems enabling combination therapy. (A) SMTA enhances therapeutic efficacy through PDT effects, delivering chemo-agents, and activating the cGAS-STING pathway. (B) Flow cytometry and the quantitative analysis of DC maturation after various treatments. Reprinted with the permission from Ref. 286. Copyright © 2022, Wiley-VCH GmbH. (C) Design of cMn-MOF@CM combining nanovaccine and biomimetic SDT effects. (D) DC maturation and T cell activation induced by cMn-MOF@CM. (E) Tumor growth curves of primary tumors and distant tumors. Reprinted with the permission from Ref. 290. Copyright © 2021, Elsevier B.V.

The external energy responsiveness of metallic nanocarriers can be leveraged as a significant advantage in combination therapy, offering a key benefit over conventional organic nanocarriers^103^^,^^121^^,^^283^^,^^284^. Wang et al.^285^ demonstrated the potentiation of immune responses by conjugating BODIPY onto platinum nanoparticles. The initial release of BODIPY from the nanoparticles triggered the first wave of PDT upon laser irradiation, while subsequent platinum release induced ER stress and extended the PDT effect independent of light irradiation. Consequently, these nanocomplexes elicited stronger and more durable immune responses than single PDT agents, nearly eradicating triple-negative breast cancer tumors in murine models. Zhao et al.^286^ engineered a nanoscale MOF nanoparticle by linking Mn^3+^ and porphyrin (TCPP) through coordination bonds (Fig. 6A and B). After cellular uptake, PIS reacted with intracellular GSH through a redox reaction, releasing TCPP and Mn^2+^ reduced from Mn^3+^. This transformation stimulated the cGAS–STING pathway for DC activation and synergized with TCPP-induced PDT to enhance antitumor immunity.

Liquid metals such as gallium (Ga) are emerging as a new class of functional metallic nanocarriers due to their fluidity and high photothermal conversion efficiency^287^. Qi et al.^288^ employed a simple pulse-type sonication approach to fabricate injectable liquid metal nanoparticles with combined PTT and immunomodulating functions. The liquid nanoparticle comprised an eutectic gallium-indium alloy and imiquimod in a core-shell structure, and selectively accumulated in tumor tissues after intravenous injection. Under laser irradiation, they induced potent PTT effects for tumor ablation, meanwhile remotely releasing imiquimod to activate TLR signaling. Such synergistic activities promoted the infiltration and activation of DCs and cytotoxic T cells in targeted tumors over a 14-day period for enhanced cancer immunotherapy.

In addition to photo-triggered therapies, metallic nanomedicines are also widely employed in SDT due to the metal cluster charge transfer mechanism^289^. Zhan et al.^290^ integrated a manganese-based MOF with CpG and tumor cell membranes to form cMn-MOF@CM. This nanoplatform exhibited prolonged blood circulation and enhanced tumor targeting efficiently *via* EPR effects. Upon ultrasound stimulation, cMn-MOF@CM induced significant STD effects while triggering cargo release (Fig. 6C–E). The tumor antigens, both *in situ* derived from SDT and from the coating cell membrane, initiated vaccine-like responses, further synergizing with the immune adjuvant CpG to promote DC maturation and T cell activation. This strategy proved effective against both local primary tumors exposed to ultrasound and untreated distant tumors.

As the field of metallography develops, an increasing number of functional metal-based substances have been developed for use as both drug carriers and immunological agents, creating a versatile platform that can help overcome drug resistance and enhance therapeutic efficacy^291^. While much attention has been given to their multifunctional properties, comparatively little focus has been placed on the critical role of dosing and timing schedules in these combination therapies. Moreover, the ADME processes of these metal nanoparticles remain poorly understood, and undesired tissue accumulation often leads to side effects and toxicity^292^. A recent study identified a previously unrecognized mechanism through which mitotically quiescent proximal tubular epithelial cells eliminate the endocytosed gold nanoparticles and even partially transformed into large nanoassemblies within lysosomes and endosomes^293^. These gold-containing endocytic vesicles were then squeezed off the membranes and released into the lumen, allowing the proximal tubular epithelial cells to eliminate more than 95% of the endocytosed gold nanoparticles from the kidneys into the urine within a month. This finding provides valuable insights into the excretion of gold nanoparticles and highlights potential long-term safety risks associated with their accumulation and organelle-damage in the body.

## 5 Metallic nanomedicines in oncology clinical trials and their immunological insights

Immunotherapeutic approaches, such as checkpoint inhibitors, have fundamentally transformed clinical cancer treatment^294^. A completed phase I clinical trial (NCT03991559) investigated the combination of PD-1 checkpoint blockade with Mn^2+^ for the treatment of twenty-two patients with advanced metastatic solid tumors. This combination demonstrated promising clinical efficacy, achieving a 45.5% best objective response rate^22^. Mechanistic studies using peripheral blood mononuclear cells from these patients revealed a significant correlation between clinical response and the level of Mn^2+^-induced STING activity. Notably, disease control was achieved in all five patients who had previously shown resistance to PD-1 antibody combined with chemotherapy or radiotherapy, including three with partial response and two with stable disease. No Mn^2+^-related toxicity or accumulation in the basal ganglia was observed. This phase I study provided compelling evidence supporting the biosafety and therapeutic efficacy of Mn^2+^, prompting the initiation of a phase II study. The therapeutic potential of Mn^2+^-induced STING activation in the treatment of other types of tumors, including biliary tract cancer, ovarian cancer, and lymphoma, is currently being evaluated (NCT04004234, NCT03989336, and NCT04873440). In addition to their inherent immune modulating functions, metallic nanomedicines offer advantages such as passive accumulation in solid tumors *via* the EPR effect and sharp responsiveness to external energy sources, enhancing the efficacy of phototherapy or radiotherapy^266^. The following sections summarize key clinical studies of metallic nanomedicines in cancer treatment, highlighting their role as either delivery vehicles or inducers of RCD in immunotherapy (Table 2).

**Table 2:** Clinical trials of metallic nanomedicine in cancer therapy.

Nanoparticle	Metal	Indication	Clinical trial	Phase	Participant	Start date
CNSI-Fe(II)	Fe	Advanced solid tumors	NCT06048367	I	24	03/2020
SPION/SMF	Fe	Osteosarcoma	NCT04316091	I	60	09/2020
ZnO-Fe_3_O_4_	Zn, Fe	Oral potentially malignant lesions	NCT06271564	I	20	02/2024
CYT-6091	Au	Advanced solid tumors	NCT00356980	I	60	05/2006
NU-0129	Au	Glioblastoma multiforme	NCT03020017	I	8	05/2017
CD24-Gold	Au	Salivary gland tumors	NCT04907422	NA	60	08/2018
Cavrotolimod	Au	Advanced solid tumors	NCT03086278	I	16	10/2017
	Au	Advanced solid tumors	NCT03684785	I/II	57	12/2018
AuroShell	Au	Head and neck cancer	NCT00848042	NA	11	04/2008
NC-6004	Pt	Advanced solid tumors	NCT02240238	II	209	05/2014
NBTXR3	Hf	Pancreatic cancer	NCT04484909	I	24	07/2020
	Hf	Oesophageal cancer	NCT04615013	I	24	11/2020
	Hf	Non-small cell lung cancer	NCT04505267	I	24	02/2021
	Hf	Head and neck squamous cell	NCT04892173	III	500	01/2022
RiMO-301	Hf	Prostate adenocarcinoma	NCT02805894	I/II	5	11/2017
	Hf	Advanced solid tumors	NCT03444714	I	18	04/2018
	Hf	Head and neck cancer	NCT05838729	I/II	16	04/2023
AGuIX	Gd	Gynaecological cancer	NCT03308604	I	18	05/2018
	Gd	Lung tumors and pancreatic cancer	NCT04789486	II	100	05/2021
	Gd	Brain tumor and metastasis	NCT04899908	II	134	09/2021
	Gd	Brain tumor and metastasis	NCT04881032	I/II	66	03/2022

### 5.1 Gold nanoparticles

The adoption of metallic nanoparticles in clinical cancer therapy has greatly gained public attention. Notably, metallic nanoparticles comprised 11% of overall nanomedicine entering clinical trials between 2016 and 2021^295^. The first metallic immune-relevant nanomedicine to enter clinical evaluation was CYT-6091 (Aurimune) in 2006. This treatment employed 27 nm PEG-functionalized gold nanoparticles to deliver recombinant human TNF-*α* as an immunotherapeutic agent to twenty-nine patients with advanced cancer. The nanoscale size of CYT-6091 facilitated its accumulation in tumor tissues through the EPR effect, while the PEG coating helped shield it from rapid clearance by the reticuloendothelial system. Pharmacokinetic data indicated a roughly 5-fold increase in the circulating half-life of CYT-6091 compared to soluble TNF-*α*. Biopsy samples showed preferential accumulation of CYT-6091 in tumor tissues, with minimal presence in healthy organs including the liver. Notably, CYT-6091 mitigated dose-limiting toxicities such as hypotension, hepatotoxicity, malaise, and fatigue, which are commonly associated with TNF-*α* alone. Phase II studies are planned for pancreatic cancer patients in combination with second-line therapies, though further details are yet to be announced. CYT-21000 was a second-generation Aurimune platform that carried both TNF-*α* and paclitaxel. In this formulation, TNF-*α* disrupted tumor vasculature, facilitating paclitaxel penetration into the tumor tissue for a synergistic antitumor effect. Similarly, CYT-61000 and CYT-71000 gold nanoparticles are designed to deliver interferon and gemcitabine, respectively.

Inspired by the effective tumor penetration and favorable biosafety profile of the CYT series, several other gold-based nanomedicines have also been developed^296^^,^^297^. AuroShell, consisting of a gold shell with a thickness of 10 to 20 nm deposited on a silicon dioxide core, has entered clinical trials by the FDA for photothermal ablation of solid tumors. It demonstrated superior tumor accumulation with minimal deposition in healthy tissues following systemic administration. When illuminated with a near-infrared light source, these nanoparticles absorbed and converted the light into heat, resulting in selective PTT effects against tumor without affecting normal tissues. Remarkably, thirteen out of the fifteen prostate cancer patients treated with AuroShell exhibited no detectable signs of cancer in the target ablation zone upon biopsy, up to one-year post-treatment. This outcome may be attributed to the immune memory induced by PTT, although the underlying mechanism remains to be elucidated. Clinical trials involving patients with lung cancer and head and neck cancer are currently underway. The safety profile of AuroShell particles in clinical trials was deemed acceptable, with minimal particle-related toxicity observed based on hematology and urine analysis. However, it is important to note that there was no significant reduction in the gold content in the body following a single intravenous injection, even after one year. This raises concerns about the potential long-term risk of tissue accumulation.

Kumthekar et al.^298^ developed a nucleic acid antitumor vaccine (NU-0129) based on gold nanoparticles to overcome the challenges posed by blood–brain and blood–tumor barriers. NU-0129 comprised spherical gold nanoparticles covalently linked with siRNA oligonucleotides targeting the glioblastoma oncogene Bcl2Like12 (Bcl2L12). After intravenous injection, NU-0129 effectively reached patient brain tumors due to its ultra-small size, with gold enrichment observed in the tumor-associated endothelium, macrophages, and tumor cells. Cellular uptake of NU-0129 in glioma cells correlated with a reduction in Bcl2L12 protein expression, as indicated by comparing matched primary tumors and NU-0129-treated recurrent tumors. While the exact mechanism of NU-0129 uptake was not fully understood, over 40% of the total gold content was still detectable in tumors up to 174 days after NU-0129 treatment. Biosafety studies confirmed that NU-0129 was well tolerated, although potential long-term toxicity remained to be addressed in follow-up clinical trials.

Cavrotolimod (AST-008) consists of nucleic acids arranged on the surface of spherical gold nanoparticles. It features a CpG oligonucleotide modification that specifically activates TLR9, triggering a robust immune response. These nanoparticle-based structures enter cells more efficiently than conventional oligonucleotide analogues and do so *via* endosomal pathways, making them ideal for interacting with intracellular targets located in endosomes. In the first-in-human Phase I study, Cavrotolimod demonstrated favorable tolerability and promising pharmacodynamic properties. It is being developed for the treatment of advanced solid tumors in combination with PD-1 blockade. This combination therapy enhances circulating levels of Th1-type cytokines, along with activated peripheral T cells and NK cells. However, the results of this trial have not yet been published^299^^,^^300^.

### 5.2 Iron nanoparticles

Iron oxide nanoparticles are among the most extensively studied inorganic particles for biological applications due to their innate magnetic responsiveness and favorable biocompatibility^301^. Most approved iron oxide nanoparticles are primarily intended for diagnosing various pathologies. Among these, ferumoxytol, a 30 nm carboxymethyl dextran-coated iron oxide nanoparticle initially developed as an MRI contrast agent, has been approved for iron supplementation in treating anemia and chronic kidney disease^302^. Interestingly, ferumoxytol is also widely investigated in preclinical studies for numerous other applications, including cancer treatment. In humans, ferumoxytol has demonstrated significant preferential uptake in various solid tumors. Recent research has highlighted ferumoxytol's ability to enhance caspase-3 activity and induce TAM polarization towards the M1 phenotype, potentially fostering an immunogenically active TME. Ferumoxytol has also been shown to affect the viability and migration of DCs at high concentrations^303^. Additionally, emerging evidence reveals that the surface coating and charge of iron nanoparticles significantly impact their immune effects. For example, dextran-coated iron nanoparticles have been reported to inhibit the proliferative activity of T lymphocytes^304^. Positively charged ones enhance antigen cross-presentation by DCs, while negatively charged ones suppress DC function and rapidly activate the autophagy pathway^305^. However, these studies are currently in the preclinical stages, and the clinical translation potential of ferumoxytol in cancer therapy remains to be fully explored.

Nanocarbon iron suspension injection (CNSI-Fe) is a next-generation nanomedicine developed based on carbon nanoparticle suspension, with Fe^2+^ serving as the antitumor active ingredient and nano charcoal acting as the carrier for Fe^2+^. In the preparation, numerous oxygen-containing groups on CNSI interacted with Fe^2+^, facilitating the formation of a nanoscale complex. This nanocomplex exhibited rapid and specific accumulation of Fe^2+^ in tumor cells without causing systemic toxicity in animal models^306^. It effectively catalyzes the Fenton reaction and induces ICD effects on tumor cells through ferroptosis. A phase I clinical trial (NCT06048367) assessed the safety, tolerability, pharmacokinetics profile, and preliminary efficacy of CNSI-Fe in patients with advanced solid tumors in 2020, though results remain undisclosed. Currently, CNSI-Fe is set to enroll 54 patients in a Phase Ib/IIa clinical trial (CTR20243192) to evaluate its therapeutic efficacy in treating advanced solid tumors.

### 5.3 Other metallic nanomedicines

The FDA approval of cisplatin in 1978 marked a significant milestone in the exploration of metal complexes for biological and medical applications. Among metallodrugs, platinum-based therapies have stood out in clinical oncology, retaining their efficacy as first-line treatments despite challenges such as drug resistance and side effects. NC-6004, a polymeric micelle that encapsulated cisplatin using amphiphilic PEG_12000_-*b*-P(Glu)_6000_, demonstrated a particle size of 28 nm and an impressive drug loading capacity of up to 39%^307^. It remained stable and could release cisplatin over 150 h in saline solution. In clinical trials, NC-6004 combined with gemcitabine for treating advanced solid tumors, particularly pancreatic cancer, achieved a remarkable disease control rate of 64.7% with well-tolerated doses. Systemic toxicity associated with cisplatin was observed only at doses up to 120 mg/m^2^ ^308^^,^^309^. Phase III clinical trials for treating advanced or metastatic pancreatic cancer have been completed. In another phase II study combining NC-6004 with gemcitabine for patients with unresectable cancers, disease control rates were notable: 35.3% in non-small cell lung cancer, 38.0% in biliary tract cancer, and 53.8% in bladder cancer cases. Recent findings suggest that low doses of platinum drugs can activate the cGAS–STING signaling pathway by inducing breaks in nuclear dsDNA, thereby enhancing both innate and adaptive antitumor immune responses. This underscores the potential of platinum drugs to treat certain cancers at tolerable doses without causing systemic toxicity, and highlights promising combinations with other immunotherapeutic approaches.

RiMO-301, a Hf-based MOF nanoparticle designed to potentiate radiation therapy and checkpoint blockade immunotherapy, represents the first MOF to enter clinical trials. When combined with αPD-1 antibodies, RiMO-301 achieved an objective response rate of 66.7% (4 out of 6 patients), with the remaining 2 patients showing a 15%–20% reduction in tumor size. Observed treatment-related adverse events included mild radiation dermatitis, dysgeusia, hypotension, dizziness, and elevated aminotransferase levels^310^. Based on its promising performance in patients with advanced solid tumors, RiMO-301 has been approved to advance to Phase 2 clinical studies.

Another metallic nanoparticle under clinical development is NBTXR3, designed as a radiation enhancer comprising negatively charged phosphate-coated crystalline hafnium oxide nanoparticles^311^. This formulation exhibited exceptional X-ray absorbance and demonstrated a favorable safety profile *in vivo*. Upon a single intratumoral injection, these nanoparticles can be activated by RT, leading to massive tumor cell death. While the tumor ablation induced by NBTXR3 does not constitute a form of immunotherapy per se, recent studies indicated that the release of antigens from irradiated tumor cells could prime the immune system, triggering systemic and durable antitumor immunity. Therefore, further investigation into the immunological implications of this nanoparticle is warranted. Harnessing this physical mode of action, NBTXR3 showed promise for treating various solid tumors amenable to RT, especially in combined treatment approaches such as with immune checkpoint inhibitors. In a randomized controlled trial comparing NBTXR3 combined with RT *vs.* RT alone for locally advanced soft tissue sarcoma, pathological complete remission was observed in fourteen cases (16%) in the NBTXR3 group compared to seven cases (8%) in the RT alone group. The most common grade 3–4 treatment-emergent adverse event associated with NBTXR3 was hypotension (7%, six out of eighty-nine patients)^312^. Currently, NBTXR3 is approved and available in Europe for treating patients with soft tissue sarcomas^313^.

Taken together, metallic nanomedicines hold significant promise in enhancing antitumor immune responses, either by directly activating innate immune pathways such as cGAS–STING or by potentiating PTT or RT-induced tumor ablation and subsequent ICD effects. These approaches offer advantages in inducing the abscopal effect and promoting immune memory. However, most metallic nanomedicines remain in preclinical or early-stage trials. A deeper understanding of immune network interactions and comprehensive investigations into the short-term and long-term toxicity of metal-based materials are crucial prerequisites for advancing clinical translation.

## 6 Conclusions and future prospects

Immunotherapy leverages the host's immune system to attack tumor cells, representing a significant advancement in cancer treatment over the past decade^12^. Emerging studies have revealed that metal ions can modulate the dynamics of cancer immunity by directly or indirectly impacting biological activities across diverse cell populations. Directly, they stimulate immune cells through pathways such as cGAS–STING, TLR, NF-*κ*B, and NLRP3 in myeloid cell populations, as well as TCR, LFA-1, and NFAT signaling cascades in lymphocytes. These effects lead to a reduction in MDSCs, a shift in TAMs from an M2 to M1 phenotype, enhanced maturation and antigen presentation capabilities of DCs, and the priming of antigen specific CD8^+^ T cells. Indirectly, metal ions influence the TME by disrupting the ECM or addressing immunosuppressive features such as acidity and hypoxia. These actions not only promote tumor cell growth and metastasis but also hinder immune cell infiltration, activation, and cytotoxic function. Additionally, metal ions can trigger various RCD patterns of tumors, which subsequently enhance the antigenicity and immunogenicity of the TME, further supporting immune responses.

While direct effects on immune cells are rapid and specific, indirect effects on tumor cells and the TME tend to be more gradual and may vary across individuals or even different stages of tumor progression within the same individual. This underscores the importance of personalized assessment and tailored therapy. Nonetheless, these indirect actions often contribute to a more favorable environment for immune training, systemic immune protection, and the establishment of immune memory. Combining both direct and indirect mechanisms may overcome potential negative feedback loops that contribute to tumor resistance, generating both rapid and sustained antitumor effects, ultimately improving response rates and providing survival benefits. However, the presence of multiple cell types within tumor tissues and the shared immune signaling pathways across these cells pose challenges in isolating these effects. For example, toxic ions used to initiate RCD in tumor cells may inadvertently damage healthy endothelial and immune cells. Additionally, cGAS–STING activation in T cells is often associated with unwanted apoptosis, which could suppress antitumor immunity^314^. Therefore, a cell-type-specific targeting strategy with spatiotemporal precision could offer insights into these challenges and enhance the efficacy of oncotherapy.

Exposure to high levels of metal ions poses severe biosafety concerns such as hematologic toxicity and organ failure^315^. For instance, excess iron can cause oxidative damage in cardiomyocytes, while elevated copper levels may increase oxidative stress in pancreatic *β* cells^316^^,^^317^. Additionally, systemic administration of Mn^2+^ may introduce the risk of STING-related cytokine storms, which can lead to autoimmune diseases due to excessive immune system activation. Nanotechnology offers a promising solution by enabling precise delivery of metal ions, either as integrated nanocomplexes or encapsulated cargos, across physiological and pathological barriers to specific tissues, cells, and organelles^318^^,^^319^. The biodistribution of metallic nanomedicines can be tailored based on their physicochemical properties and administration routes. For example, particles sized between 10–50 nm can passively drain to lymph nodes following subcutaneous injection, while those sized between 10–200 nm normally accumulate in solid tumor sites *via* leaky vasculature after intravenous injection^320^^,^^321^. Other factors such as shape, electric charge, stiffness, and surface coating also play crucial roles in determining the ADME processes of nanomaterials^322^. Therefore, a rationally designed architecture of these nanomaterials could potentially advance their reality of cancer therapy. Moreover, stimuli-responsive nanostructures designed to respond to pH, oxidative stress, or enzyme expression variations in different environments offer a customized approach to trigger cargo release at the intended sites^227^^,^^323^.

Despite promising, only a limited number of metallic nanomedicines have entered clinical evaluation. Several challenges need to be addressed to facilitate their translation. First, the pharmacokinetics and biocompatibility of metallic nanomedicines remain inadequately studied. Growing evidence suggests that metallic nanoparticles entering the body are prone to deposit in the liver, spleen, and kidneys, potentially inducing oxidative stress and increasing inflammation^324^^,^^325^. Consequently, they damage biomolecules such as proteins, nucleic acids, and lipids ^326^, contributing to the onset of various diseases, including diabetes, neurodegenerative disorders, cardiovascular diseases, and even cancer itself. For example, high amounts of TiO_2_, Au, and Ag nanoparticles can induce endothelial leakiness by promoting gaps between endothelial cells^327^^,^^328^. This effect accelerates the intravasation and extravasation of breast cancer cells, thereby increasing the extent of existing metastasis and facilitating the emergence of new metastatic sites. Additionally, CuO nanoparticles have been demonstrated to disrupt embryonic development by interfering with mitochondrial autophagy and inhibiting the tricarboxylic acid cycle^329^. The physicochemical characteristics of metallic nanoparticles may greatly influence their safety profile. Studies indicate that smaller silver nanoparticles (10 nm) demonstrate greater permeability into mitochondria and nuclei compared to larger counterparts (40 or 100 nm), ultimately causing severe hepatobiliary toxicity^330^. The renal clearance efficiency of positive charged gold nanoparticles is approximately 2.5 times lower than their negative-charged counterparts^293^. Therefore, it is essential to understand the parameters such as size, charge, surface property, and chemical composition that impact the *in vivo* behavior of metallic nanoparticles. There is also a need for more specific indicators related to metal-induced pathological features, such as organelle dysfunction, oxidative stress, nerve injury, chronic autoinflammation, and long-term genotoxicity^331^. A large-scale machine learning analysis of metallic nanoparticles in preclinical and clinical cancer research could provide evidence to support standardizing their use in this context.

Second, comprehensive mechanistic investigations into the interactions between metal substances and immune networks, along with strategies for precise control over delivery and release, are essential. Certain metal ions may exhibit multifaced bioactivities^332^. For example, Mn^2+^ has been shown to activate both the NLRP3 inflammasome and the cGAS–STING signaling pathways^333^. However, it remains unclear which pathway plays a predominant role in Mn^2+^-induced immunological functions, and whether the effective dose threshold varies between them. Moreover, the effects are likely dependent on the specific cell types involved. Activation of NLRP3 within TAMs can lead to cell repolarization, whereas its activation in tumor cells can induce pyroptosis^334^^,^^335^. Similarly, cGAS–STING signaling in DCs promotes cell maturation, whereas in T cells, its activation is often associated with apoptosis^314^. Undesirable stimulation may not only compromise antitumor efficacy but also trigger negative feedback mechanisms that promote tumor growth. In clinical applications, cancer patients often present with various complications, and metal nanoparticles can activate multiple immune signaling pathways, offering additional functions such as anti-infection effects^336^. In short, the complexity of interactions between metal nanoparticles and diseases highlights the need for further exploration of these underlying mechanisms.

Third, compared to small-molecule drugs, metallic nanomedicines introduce structural complexity, adding costs and steps in chemistry, manufacturing, and controls^337^. Therefore, robust synthesis strategies and analytical methods must be established early in the development process to facilitate scaling-up and translation into clinical practice. Last, there are challenges due to the lack of comprehensive regulatory guidance, as only a few candidates have progressed to clinical trials. The high costs of developing new formulations, combined with the absence of approved precedents, may deter investigators from pursuing clinical translation. Indeed, many leading research groups focusing on clinical applications have redirected their attention to non-metallic particles for cancer immunotherapy. Overcoming these obstacles will be crucial for realizing the full potential of metallic nanomedicines in making a clinical impact on public health.

## Author contributions

Shixuan Li and Xiaohu Wang collected the related papers, drafted the original manuscript. Huiyun Han, Shuting Xiang, Mingxi Li, Guangyu Long, Yanming Xia collected the related papers and revised the manuscript. Qiang Zhang and Suxin Li outlined and finalized the manuscript. All of the authors have read and approved the final manuscript.

## Conflicts of interest

The authors declare no conflict of interest.
